# Risk factors for early recurrence in patients with hormone receptor-positive, HER2-negative breast cancer: a retrospective cohort study in Japan (WJOG15721B)

**DOI:** 10.1007/s12282-025-01700-y

**Published:** 2025-04-10

**Authors:** Rurina Watanuki, Hitomi Sakai, Yuri Takehara, Atsushi Yoshida, Naoki Hayashi, Yukinori Ozaki, Akemi Kataoka, Natsue Uehiro, Hidenori Kamio, Mai Onishi, Atsushi Fushimi, Takashi Ikeno, Masashi Wakabayashi, Mayumi Iida, Tsutomu Kawaguchi, Toshimi Takano

**Affiliations:** 1 Department of Breast Surgery, National Cancer Center Hospital East, 6-5-1 Kashiwanoha, Kashiwa-shi, Chiba 277-8577 Japan; 2 Advanced Cancer Translational Research Institute, Showa Medical University, 1-5-8 Hatanodai, Shinagawa-ku,, Tokyo 142-8555 Japan; 3 Department of Breast Surgical Oncology, St. Luke’s International Hospital, 9-1 Akashicho, Chuo-ku, Tokyo 104-8560 Japan; 4 Division of Breast Surgical Oncology, Department of Surgery, Showa University, 1-5-8 Hatanodai, Shinagawa-ku, Tokyo 142-8555 Japan; 5 Breast Oncology Center, Cancer Institute Hospital of the Japanese Foundation for Cancer Research, 3-8-31 Ariake, Koto-ku, Tokyo 135-8550 Japan; 6 Department of Breast Surgery, Tokyo Metropolitan Cancer and Infectious Diseases Center Komagome Hospital, 3-18-22 Honkomagome, Bunkyo-ku, Tokyo 113-8677 Japan; 7 Department of Medical Oncology, National Cancer Center Hospital, 5-1-1 Tsukiji, Chuo-ku, Tokyo 104-0045 Japan; 8 Department of Surgery, The Jikei University School of Medicine, 3-19-18 Nishi-Shinbashi, Minato-ku, Tokyo 105-8471 Japan; 9 Clinical Research Support Office, National Cancer Center Hospital East, 6-5-1 Kashiwanoha, Kashiwa-shi, Chiba 277-8577 Japan; 10 Biostatistics Division, Center for Research Administration and Support, National Cancer Center, 6-5-1 Kashiwanoha, Kashiwa-shi, Chiba 277-8577 Japan; 11 Japan Drug Development and Medical Affairs, Eli Lilly Japan, 5-1-28 Isogamidori, Chuo-ku, Kobe-shi, Hyogo 651-0086 Japan

**Keywords:** HR-positive HER2-negative breast cancer, Early recurrence, Risk factor, Nomogram

## Abstract

**Background:**

Patients with early recurrence of hormone receptor (HR)-positive, HER2-negative (HR+/HER2−) breast cancer have a poor prognosis. We aimed to identify clinical and pathological risk factors for recurrence within three years after surgery of HR+/HER2− breast cancer.

**Methods:**

We retrospectively reviewed clinical data of patients with stage II–III HR+/HER2− breast cancer who received adjuvant endocrine therapy from January 1, 2012 to January 1, 2017 at five institutions. Using univariable and multivariable analyses, we determined risk factors for invasive disease-free survival (IDFS). A nomogram was generated using variables from the multivariable analysis to predict 3-year IDFS rate.

**Results:**

A total of 2732 patients were analyzed, with a median follow-up of 7.1 years. The 3-year IDFS rate was 92.1%. Multivariable analysis for IDFS revealed significant risk factors: age (40–69 vs. 20–39 years: HR 0.69, *p* = 0.011), nuclear grade (Grade 2 vs. Grade 1: HR 1.66, *p* < 0.001; Grade 3 vs. Grade 1: HR 1.64, *p* < 0.001), vascular invasion (Yes vs. No: HR 1.36, *p* = 0.027), pathological invasive tumor size (2–5 cm vs. < 2 cm: HR 1.75, *p* < 0.001; ≥ 5 cm vs. < 2 cm: HR 2.07, *p* < 0.001), number of positive lymph nodes (≥ 4 vs.0: HR 1.70, *p* < 0.001), and neoadjuvant chemotherapy (NAC) (Yes vs. No: HR 2.41, *p* < 0.001). The nomogram’s concordance index was 0.68.

**Conclusion:**

Younger age, nuclear grade, vascular invasion, tumor size and number of lymph node metastases were identified as independent risk factors for early recurrence. Patients whose physicians chose NAC had worse survival than those who did not.

**Supplementary Information:**

The online version contains supplementary material available at 10.1007/s12282-025-01700-y.

## Introduction

The hormone receptor-positive, human epidermal growth factor receptor 2-negative (HR+/HER2−) subtype accounts for 70% of breast cancer cases [1]. Although patients with HR+/HER2− breast cancer generally have a good prognosis, there are high-risk patients who need intensive treatment. Early recurrence occurring during the first 2–3 years of adjuvant endocrine therapy in HR+/HER2− breast cancer is considered primary endocrine resistance, resulting in a poor prognosis [2].

A new adjuvant therapy is available to reduce the risk of early recurrence of high-risk disease. In monarchE, a randomized phase III trial that investigated the addition of abemaciclib to endocrine therapy in patients with HR+/HER2−, node-positive, high-risk early breast cancer, a compilation of clinicopathological factors including nodal status, tumor size, grade, and Ki-67 were used to define “high risk” [3]. The study was aimed to treat patients with primary endocrine-resistant breast cancer who were likely to experience recurrence within the first five years.

The addition of abemaciclib significantly reduced the risk of developing invasive disease-free survival (IDFS) event [hazard ratio (HR) = 0.71, 95% confidence interval (CI) 0.58–0.87, *p* < 0.001], and this benefit was maintained after the completion of 2-year abemaciclib treatment [HR = 0.70, 95% CI 0.59–0.82, *p* < 0.001, IDFS rates by 3 years: abemaciclib plus endocrine therapy group versus endocrine therapy; 88.8% versus 83.4%]. As the population meeting all the inclusion criteria for the monarchE trial was extremely high-risk for early recurrence, we hypothesized that there may be other high-risk cohorts that do not meet all of the inclusion criteria of monarchE. They may need more intensive adjuvant therapies (e.g., abemaciclib) than the current standard of care, which includes adjuvant endocrine therapy without chemotherapy.

The present retrospective study (Retrospective multi-center observational study to explore the risk-factor for early recurrence in HR+/HER2− Early breast cancer: RealisE study; WJOG15721B) aimed to identify risk factors for early recurrence within three years after surgery by regressing early recurrence on clinicopathological characteristics, perioperative treatment in patients with early-stage HR+/HER2− breast cancer.

## Patients and methods

### Data source and collection

This was a retrospective cohort study. We retrieved clinicopathological and survival data of eligible patients from breast cancer databases of five Japanese institutions (supplementary appendix) which are members of the West Japan Oncology Group (WJOG). The original databases of these participating hospitals contained both structured and unstructured data from medical records, and investigators at these hospitals manually extracted data of interest, which included age at diagnosis; comorbidities; menopausal status; presence of bilateral breast cancer; clinical and pathological stage (UICC TNM classification 7th or 8th edition); tumor characteristics including histological type, histological grade, nuclear grade, estrogen receptor (ER) status, progesterone receptor (PgR) status, and HER2 status; Ki-67 index; lymphatic invasion; vascular invasion; pathological invasive tumor size; number of pathological positive lymph nodes; and therapeutic response. Details of treatment, survival data, and recurrence patterns were also extracted.

The study protocol was approved by the Showa University Research Ethics Review Board (22-017-B). Data cut-off date was November 2,2022.

### Patients

**Fig. 1:**
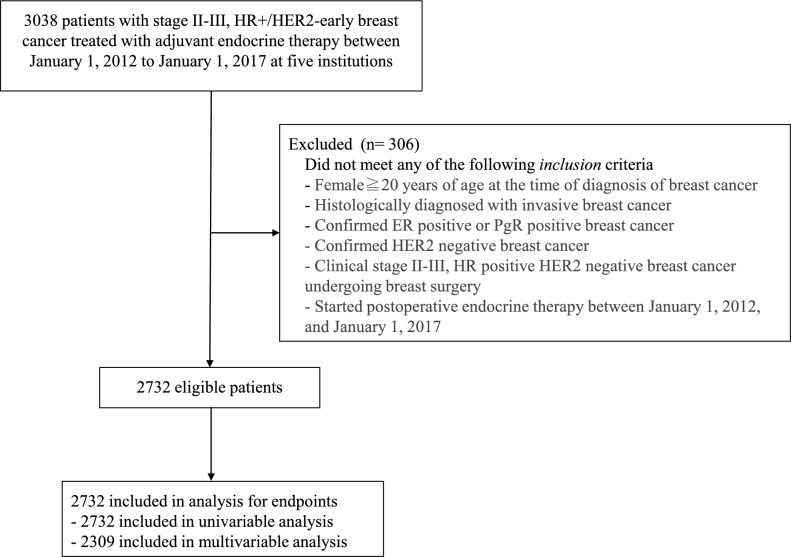
Patient flow diagram. *ER* estrogen receptor, *PgR* progesterone receptor, *HER2* human epidermal growth factor 2, *HR* hormone receptor

Female patients aged ≥ 20 years with stage II-III HR+/HER2− breast cancer were eligible. HR positivity was defined as having ≥ 1% expression of either ER or PgR, or an Allred score ≥ 3, by immunohistochemistry (IHC). Tumors were classified as HER2− based on the American Society of Clinical Oncology (ASCO) /College of American Pathologists (CAP) guideline [4]. Both IHC and in situ hybridization (ISH) testing for gene amplification to determine HR and HER2 status were performed as part of standard clinical care. Patients were considered eligible for the study even if ISH was not performed, provided that HER2 positivity (IHC staining 2+ and ISH positive or 3+) was not observed in the tumor before or after surgery. Patients who started adjuvant endocrine therapy from January 1, 2012 to January 1, 2017 were eligible. Any duration of endocrine therapy was acceptable, and neoadjuvant/adjuvant chemotherapy was allowed. Two investigators independently reconfirmed the eligibility of patients and developed the final dataset (Fig. 1). Patients treated with adjuvant abemaciclib or olaparib were not included because these drugs were approved in Japan after the eligibility period noted above (2021 for adjuvant abemaciclib and 2022 for adjuvant olaparib).

Information about the study and how to opt-out were provided on the institutions’ websites. Patients were included in the study unless they explicitly declined to participate or opted out.

### Endpoints

Previous studies have adopted various definitions of early recurrence such as 2.5 years after diagnosis or five years following surgery [5, 6], with no settled definition. Taking into account that primary endocrine resistance is defined as relapse during the first two years of adjuvant endocrine therapy in the European School of Oncology (ESO)- European Society of Medical Oncology (ESMO) international consensus guidelines [2], as well as the treatment benefits of abemaciclib extended beyond the 2-year treatment period in monarchE [7], we defined early recurrence as recurrence occurring within three years after surgery. The primary endpoint was the 3-year IDFS rate after surgery. Secondary endpoints were IDFS, overall survival (OS), 3-year distant recurrence-free survival (DRFS) rate. IDFS, OS, and DRFS were defined according to the Standardized Definitions for Efficacy End Points in Adjuvant Breast Cancer Trials (STEEP) criteria (Supplementary method) [8]. We defined the date of surgery as the commencement date for IDFS, OS, and DRFS.

### Statistical analysis

The target sample size was set at 3000 based on the number of breast cancer patients in the five institutions. IDFS, OS, and DRFS were estimated using the Kaplan–Meier method. To investigate prognostic factors, we used the candidate clinicopathological factors listed in Tables 1 and 2 as explanatory variables in univariable analysis and multivariable Cox proportional hazards model. Explanatory variables were selected based on previous studies and medical evidence, which could be risk factors for early recurrence in patients with HR+/HER2− breast cancer. Missing values were not imputed and were categorized as ‘unknown’. For patients with bilateral breast cancer, clinicopathological factors of the side of the breast deemed prognostically worse were used for univariable and multivariable analyses. Two investigators independently chose the side of the breast and matched their individual responses. To develop a prediction model for IDFS, multivariable Cox proportional hazards models using stepwise selection with a significance level of 0.05 for explanatory variables was performed to generate a nomogram. Split internal validation was also performed to evaluate the model developing algorithm. Using total points calculated from the nomogram for discrimination, a Kaplan–Meier curve for IDFS stratified by scores, segregated with arbitrarily set cut-off. Additionally, a Cox proportional hazards model was generated to estimate HRs and the concordance index (C-index). Risk classifications were performed so that each group had an equal number of patients, and predictive accuracy was evaluated by a calibration curve.

**Table 1:** Characteristics of the study population

Characteristics	No. (%) (n = 2732)
Age, years	
Median (range)	51 (23–96)
Sex	
Female	2732 (100)
Performance status	
0	1318 (48.2)
1	16 (0.6)
Unknown	1398 (51.2)
Comorbidity	
None	1057 (38.7)
Hypertension	219 (8.0)
Diabetes	87 (3.2)
Malignant disease except breast cancer	59 (2.2)
Cardiac disease	16 (0.6)
Cerebrovascular and peripheral vascular disease	8 (0.3)
Collagen disease	8 (0.3)
Chronic liver disease	5 (0.2)
Renal dysfunction	4 (0.1)
Other	405 (14.8)
Unknown	1004 (36.7)
Menopausal status	
Premenopausal	1395 (51.1)
Postmenopausal	1311 (48.0)
Unknown	26 (1.0)
Diagnostic occasion	
Symptom awareness	2005 (73.4)
Detection by medical checkup	404 (14.8)
Accidental detection	94 (3.4)
Other	229 (8.4)
Bilateral breast cancer	
No	2690 (98.5)
Yes	42 (1.5)
Synchronous	42 (1.5)
Metachronous	0 (0)

**Table 2:** Clinicopathological features of the study population

	No. (%) (n = 2732)
Clinical T factor	
Tis	2 (0.1)
T1	193 (7.1)
T2	2205 (80.7)
T3	197 (7.2)
T4	135 (4.9)
Clinical N factor	
N0	1781 (65.2)
N1	775 (28.4)
N2	88 (3.2)
N3	88 (3.2)
Clinical stage	
IIA	1841 (67.4)
IIB	529 (19.4)
IIIA	160 (5.9)
IIIB	114 (4.2)
IIIC	88 (3.2)
Pathological T factor	
T0	18 (0.7)
Tis	23 (0.8)
T1	864 (31.6)
T2	1290 (47.2)
T3	257 (9.4)
T4	32 (1.2)
Unknown	248 (9.1)
Pathological N factor	
N0	1323 (48.4)
N1	995 (36.4)
N2	281 (10.3)
N3	124 (4.5)
Unknown	9 (0.3)
Pathological stage	
0	19 (0.7)
I	519 (19.0)
IIA	975 (35.7)
IIB	593 (21.8)
IIIA	374 (13.7)
IIIB	29 (1.1)
IIIC	124 (4.5)
Unknown	99 (3.6)
Histological type	
Invasive cancer	12 (0.4)
Invasive ductal carcinoma	2305 (84.3)
Special type	402 (14.7)
Invasive lobular carcinoma	165 (6.0)
Mucinous carcinoma	153 (5.6)
Invasive micropapillary carcinoma	38 (1.4)
Other	46 (1.7)
Mixed type	6 (0.2)
Unknown	7 (0.3)
Nuclear grade	
1	1041 (38.1)
2	1039 (38.0)
3	456 (16.7)
Unknown	196 (7.2)
Histological grade	
1	149 (5.5)
2	218 (8.0)
3	69 (2.6)
Unknown	2301 (84.2)
ER	
< 1%	15 (0.5)
1–9%	18 (0.7)
≥ 10%	1722 (63.0)
Unknown	977 (35.8)
PgR	
< 1%	152 (5.6)
≥ 1%	825 (30.2)
Unknown	1755 (64.2)
HER2	
0	1386 (50.7)
1+	1008 (36.9)
2+	338 (12.4)
Ki-67	
< 14%	371 (13.6)
14–29%	315 (11.5)
≥ 30%	367 (13.4)
Unknown	1679 (61.5)
Lymphatic invasion	
No	1545 (56.6)
Yes	1166 (42.7)
Unknown	21 (0.8)
Vascular invasion	
No	2427 (88.8)
Yes	284 (10.4)
Unknown	21 (0.8)
Pathological tumor size (cm)	
< 2	765 (28.0)
2 to less than 5	1423 (52.1)
≥ 5	286 (10.5)
Unknown	258 (9.4)
Number of pathological lymph node metastases	
0	1317 (48.2)
1–3	995 (36.4)
4–9	280 (10.2)
≥ 10	124 (4.5)
Unknown	16 (0.6)
Pathological therapeutic response^a^	
Grade 0	42 (5.7)
Grade 1	454 (61.6)
Grade 2	154 (20.9)
Grade 3	50 (6.8)
Unknown	37 (5.0)

*ER* estrogen receptor, *PgR* progesterone receptor, *HER2* human epidermal growth factor receptor 2

^a^Data were tabulated for the 737 patients who received preoperative chemotherapy or preoperative endocrine therapy

Cumulative incidence function (CIF) curves were used to estimate the cumulative incidence of recurrence (local recurrence or distant metastasis) and 95% CIs, treating death and second cancers as competing risks. HRs and 95% CIs were estimated by the Fine & Gray model to explore risk factors for recurrence. The cumulative incidence rate of distant metastasis and 95% CIs were estimated using the same method. All statistical analyses were performed using SAS (version 9.4).

## Results

### Patient characteristics

A flow diagram for patient selection is shown in Fig. 1. The total number of eligible patients for the final analysis was 2732. Baseline characteristics of the study population are presented in Table 1. The median age was 51 years (range 23–96), 51.5% of patients were premenopausal, and 42 patients (1.5%) had bilateral breast cancer. Clinicopathological characteristics of the eligible patients are shown in Table 2. A total of 1841 patients (67.4%) had clinical stage IIA cancer, 529 (19.4%) had stage IIB cancer, and 362 (13.3%) had stage III cancer. Missing values for histological grade were frequent. Of all patients, 1041 patients (38.1%) were found to have nuclear Grade 1 disease, 1039 patients (38.0%) had Grade 2, 456 patients (16.7%) had Grade 3, and 196 patients (7.2%) were of unknown grade. Lymphatic invasion was present in 1166 patients (42.7%) and vascular invasion in 284 (10.4%) patients. The number of pathological lymph node metastases was as follows: 1317 patients (48.2%) had none, 995 (36.4%) had 1–3, 280 (10.2%) had 4–9, and 124 (4.5%) had > 10.

Neoadjuvant chemotherapy was administered in 628 patients (23.0%) and adjuvant chemotherapy in 887 (32.5%) patients. Among these patients, most received anthracyclines or taxanes (Table 3b). Of 737 patients who received preoperative systemic therapy, 154 (20.9%) and 50 (6.8%) patients had a pathological therapeutic response of Grade 2and Grade 3, respectively (Table 2). Adjuvant endocrine therapy was administered to all patients, and 1120 (41.0%) received it for > 5 years. Details of surgical procedures and radiotherapy are shown in Table 3a.

**Table 3:** Treatment details

(a) Surgical procedures and radiotherapy	
	No. (%) (n = 2774^a^)
Surgical procedure	
Breast	
Lumpectomy	1043 (37.6)
Mastectomy	1731 (62.4)
Sentinel lymph node biopsy	
No	935 (33.7)
Yes	1839 (66.3)
Axillary lymph node dissection	
No	1452 (52.3)
Yes	1322 (47.7)
Radiation therapy	
No	1222 (44.1)
Yes	1527 (55.0)
Unknown	25 (0.9)

(b) Neoadjuvant and adjuvant endocrine therapy or chemotherapy	
	No. (%) (n = 2732)
Neoadjuvant chemotherapy	
No	2103 (77.0)
Yes	628 (23.0)
Anthracyclines	617 (22.6)
Taxanes	628 (23.0)
Others	13 (0.5)
Unknown	1 (0.04)
Neoadjuvant endocrine therapy	
No	2623 (96.0)
Yes	109 (4.0)
SERM alone	8 (0.3)
SERM + LHRH agonist	7 (0.3)
AI alone	88 (3.2)
SERM + AI	6 (0.2)
Adjuvant chemotherapy	
No	1845 (67.5)
Yes	887 (32.5)
CMF	1 (0.04)
Anthracyclines	803 (29.4)
Taxanes	887 (32.5)
Others	68 (2.5)
Adjuvant endocrine therapy	
No	0 (0)
Yes	2732 (100)
SERM alone	1002 (36.7)
LHRH alone	1 (0.04)
AI alone	1139 (41.7)
SERM + LHRH agonist	295 (10.8)
SERM → AI	177 (6.5)
SERM + LHRH agonist → AI	14 (0.5)
AI + LHRH agonist	6 (0.2)
Others	98 (3.6)
Adjuvant endocrine therapy ≥ 5 years	
No	1612 (59.0)
Yes	1120 (41.0)

*SERM* selective estrogen receptor modulator, *LHRH agonist* luteinizing hormone-releasing hormone agonist, *AI* aromatase inhibitor, *CMF* cyclophosphamide, methotrexate, and fluorouracil

^a^Total for all 2732 eligible cases; 2774 lesions including bilateral breast cancer

### Invasive disease-free survival

**Fig. 2:**
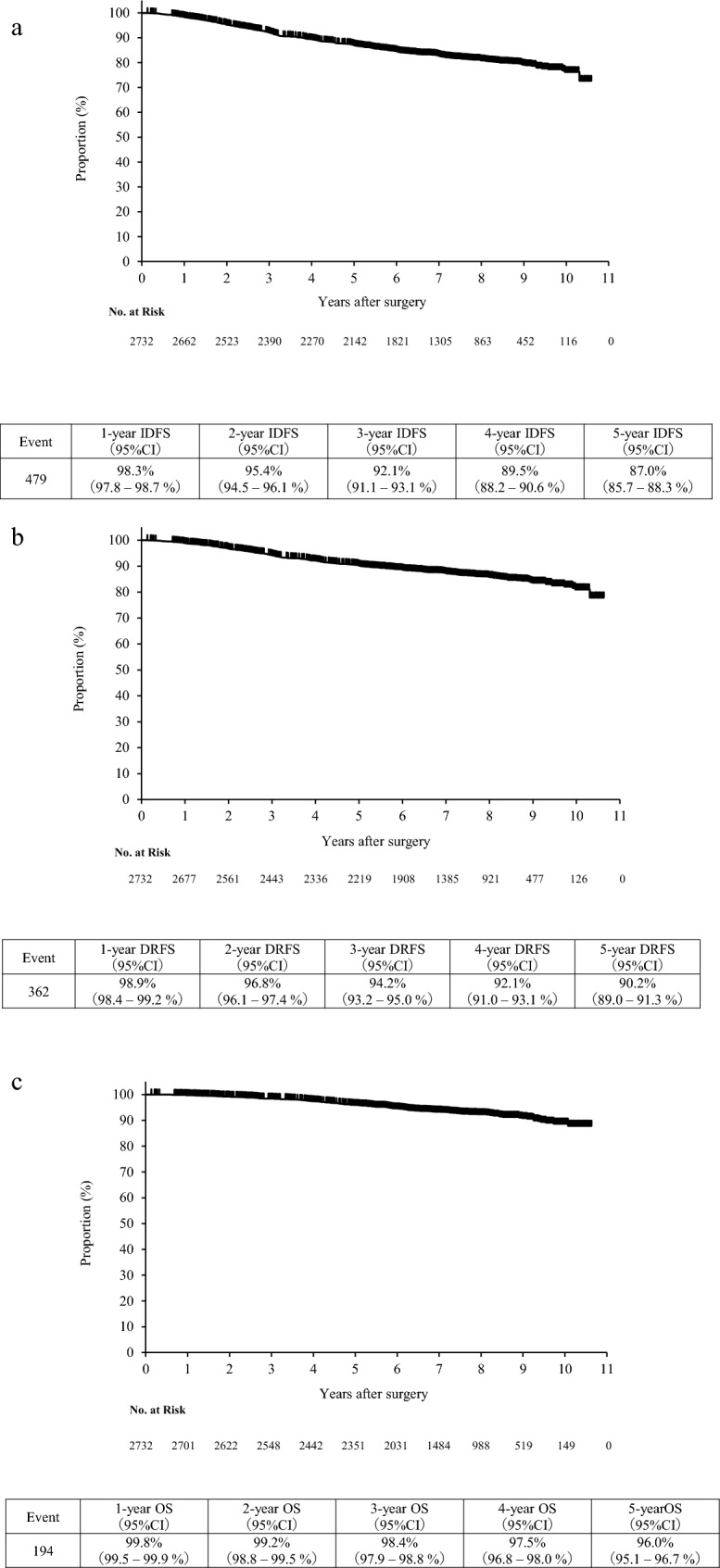
**a** Kaplan–Meier curve for IDFS, **b** Kaplan–Meier curve for DRFS, **c** Kaplan–Meier curve for OS. *IDFS* invasive disease-free survival, *DRFS* distant recurrence free survival, *OS* overall survival

The median follow-up period was 7.1 years. At the time of data cutoff, a total of 479 IDFS events were observed. The 3-year was 92.1% and the 5-year IDFS rate was 87.0% (Fig. 2a). Univariable analysis revealed that age (40–69 vs. 20–39 years: HR 0.59, 95% CI 0.46–0.77, *p* < 0.001), Ki-67 (≥ 30% vs. ≤ 14%: HR 1.90, 95% CI 1.37–2.65, *p* < 0.001), nuclear grade (Grade 2 vs. Grade 1: HR 1.84, 95% CI 1.47–2.30, *p* < 0.001; Grade 3 vs. Grade 1: HR 1.87, 95% CI 1.43–2.45, *p* < 0.001), lymphatic invasion (Yes vs. No: HR 1.67, 95% CI 1.40–2.00, *p* < 0.001), vascular invasion (Yes vs. No: HR 1.47, 95% CI 1.13–1.90, *p* = 0.003), pathological invasive tumor size (2–5 cm vs. < 2 cm: HR 1.69, 95% CI 1.31–2.15, *p* < 0.001; ≥ 5 cm vs. < 2 cm: HR 3.14, 95% CI 2.33–4.23, p < 0.001), number of pathological metastatic lymph nodes (1–3 vs.0: HR 1.44, 95% CI 1.16–1.78, *p* < 0.001; ≥ 4 vs.0: HR 2.89, 95% CI 2.29–3.62, *p* < 0.001), presence of NAC (Yes vs. No: HR 2.06, 95% CI 1.71–2.48, *p* < 0.001), and pathological therapeutic response (Grade 3 vs. Grade 0: HR 0.20, 95% CI 0.07–0.54, *p* = 0.002) were significant prognostic factors for 3-year IDFS rate (Supplementary Table 1a). There was no significant difference in 3-year IDFS rate between postmenopausal (92.8%) and premenopausal (91.5%) women (HR 1.130, 95% CI 0.944–1.352; *p* = 0.1827). The 3-year IDFS rates were 85.6%, 93.1%, and 91.6% for patients aged 20–39, 40–69, and ≥ 70 years, respectively, with a significantly worse prognosis in younger age group (40–69 vs. 20–39 years: HR 0.59, 95% CI 0.46–0.77; *p* < 0.001).

Multivariable analysis was performed on the 2309 patients for whom all explanatory variables were obtained. Results of the multivariable analysis are shown in Table 4. Factors ultimately selected for the model in the multivariable analysis were age (40–69 vs. 20–39 years: HR 0.69, 95% CI 0.51–0.92, *p* = 0.011), nuclear grade (Grade 2 vs. Grade 1: HR 1.66, 95% CI 1.31–2.11, *p* < 0.001; Grade 3 vs. Grade 1: HR 1.64, 95% CI 1.24–2.19, *p* < 0.001), vascular invasion (Yes vs. No: HR 1.36, 95% CI 1.04–1.78, *p* = 0.027), pathological invasive tumor size (2–5 cm vs. < 2 cm: HR 1.75, 95% CI 1.35–2.27, *p* < 0.001; ≥ 5 cm vs. < 2 cm: HR 2.07, 95% CI 1.48–2.89, *p* < 0.001), number of pathological metastatic lymph nodes (≥ 4 vs.0: HR 1.70, 95% CI 1.29–2.24, *p* < 0.001), and presence of NAC (Yes vs. No: HR 2.41, 95% CI 1.90–3.06, *p* < 0.001).

**Table 4:** Prognostic factors for IDFS in the multivariable analysis

Factor	Level	n (event)^a^	Including all covariates		Stepwise method (*p* = 0.05)	
			HR (95% CI)	*p* value^b^	HR (95% CI)	*p* value^b^
Age	20–39 years	233 (56)	Ref	(< 0.001)^c^	Ref	(< 0.001)
	40–69 years	1739 (279)	0.65 (0.48–0.89)	0.006	0.69 (0.51–0.92)	0.011
	≥ 70 years	337 (67)	1.06 (0.69–1.63)	0.802	1.19 (0.83–1.70)	0.359
Menopausal status	Premenopausal	1172 (199)	Ref	0.272		
	Postmenopausal	1137 (203)	1.15 (0.90–1.46)			
Bilateral breast cancer	No	2272 (392)	Ref	0.202		
	Yes	37 (10)	1.52 (0.80–2.88)			
ER	< 1%	15 (3)	Ref	(0.579)		
	1–9%	10 (3)	1.85 (0.37–9.34)	0.455		
	≥ 10%	1378 (234)	1.31 (0.41–4.26)	0.649		
	Unknown	906 (162)	1.60 (0.47–5.39)	0.449		
PgR	< 1%	137 (33)	Ref	(0.184)		
	≥ 1%	751 (146)	0.90 (0.60–1.36)	0.619		
	Unknown	1421 (223)	0.67 (0.41–1.09)	0.104		
HER2	0	1179 (217)	Ref	(0.668)		
	1+	817 (133)	1.00 (0.81–1.25)	0.977		
	2+	313 (52)	0.87 (0.64–1.19)	0.392		
Ki-67	< 14%	358 (55)	Ref	(0.782)		
	14–29%	299 (64)	1.03 (0.70–1.50)	0.889		
	≥ 30%	321 (86)	1.16 (0.78–1.73)	0.473		
	Unknown	1331 (197)	0.99 (0.70–1.42)	0.973		
Nuclear grade	Grade 1	958 (107)	Ref	(< 0.001)	Ref	(< 0.001)
	Grade 2	953 (205)	1.66 (1.30–2.13)	< 0.001	1.66 (1.31–2.11)	< 0.001
	Grade 3	398 (90)	1.50 (1.10–2.05)	0.011	1.64 (1.24–2.19)	0.001
Lymphatic invasion	No	1263 (173)	Ref	0.035		
	Yes	1046 (229)	1.29 (1.02–1.64)			
Vascular invasion	No	2035 (335)	Ref	0.153	Ref	0.027
	Yes	274 (67)	1.24 (0.92–1.66)		1.36 (1.04–1.78)	
Pathological tumor size	< 2 cm	704 (77)	Ref	(< 0.001)	Ref	(< 0.001)
	2 cm to less than 5 cm	1334 (244)	1.68 (1.29–2.19)	< 0.001	1.75 (1.35–2.27)	< 0.001
	≥ 5 cm	271 (81)	1.89 (1.34–2.66)	< 0.001	2.07 (1.48–2.89)	< 0.001
Number of pathological lymph node metastases	0	1154 (151)	Ref	(0.010)	Ref	(0.001)
	1–3	837 (153)	1.07 (0.84–1.38)	0.583	1.16 (0.92–1.46)	0.201
	≥ 4	318 (98)	1.55 (1.14–2.10)	0.005	1.70 (1.29–2.24)	< 0.001
NAC	No	1994 (290)	Ref	< 0.001	Ref	< 0.001
	Yes	315 (112)	2.21 (1.70–2.88)		2.41 (1.90–3.06)	

*IDFS* invasive disease-free survival, *CI* confidential interval, *HR* hazard ratio, *NAC* neoadjuvant chemotherapy, *Ref* reference

^a^Analysis was performed on the 2309 cases for which all explanatory variables were available

^b^Wald test p-value for HR

^c^Number of parentheses represents p-value for the comparison among all categories

Additional exploratory univariable and multivariable analyses were performed for a population of patients who did not receive NAC (Supplementary Table 1b, 1c). Multivariable analysis for IDFS in a population of patients without NAC (n = 1994) showed that nuclear glade (Grade 2 vs. Grade 1: HR 1.61, 95% CI 1.23–2.10, *p* < 0.001; Grade 3 vs. Grade 1: HR 1.59, 95% CI 1.14–2.23, *p* < 0.001)), lymphatic invasion (Yes vs. No: HR 1.32, 95% CI 1.00–1.74, *p* = 0.047), pathological invasive tumor size (2–5 cm vs. < 2 cm: HR 1.66, 95% CI 1.23–2.25, *p* = 0.001; ≥ 5 cm vs. < 2 cm: HR 2.01, 95% CI 1.31–3.06, *p* = 0.001), number of pathological metastatic lymph nodes (≥ 4 vs.0: HR 1.66, 95% CI 1.18–2.35, *p* = 0.004) were significant prognostic factors.

**Fig. 3:**
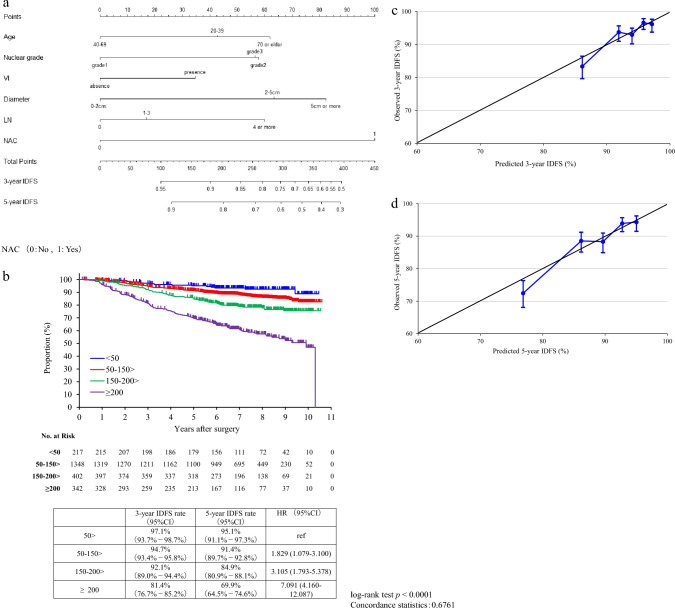
**a** Nomogram predicting 3-year IDFS rate and 5-year IDFS rate.Draw a straight line from each applicable clinical factor to the Points axis. Sum these points, locate this number on the Total points axis, and draw a straight line down to find the patient's IDFS. **b** Curve for IDFS at each point (50> , 50–150>, 150–200>, ≥ 200) based on total points calculated from the nomogram. **c** Calibration plot for predicted and observed 3-year IDFS. **d** Calibration plot for predicted and observed 5-year IDFS. The figure shows the predicted IDFS on the x-axis and the actual IDFS on the y-axis. The blue line represents the prediction by nomogram. The vertical line shows the 95% confidence interval. If this blue line is close to the diagonal of Y = X, the predictive performance is considered high. *VI* vascular invasion, *LN* lymph nodes, *NAC* neoadjuvant chemotherapy, *IDFS* invasive disease-free survival

A nomogram was generated to predict 3-year and 5-year IDFS rates using selected factors from the multivariable analysis (Fig. 3a). Based on total points calculated from the nomogram, curves for IDFS stratified by score were generated and evaluated by the log-rank test and Cox’s proportional hazards model (Fig. 3b). To test the discriminative ability of the model, we calculated the C-index, which was 0.68. In addition, risk classification was performed so that each group had an equal number of patients with respect to the total points calculated from the nomogram. Calibration plots were generated for the predicted and observed IDFS rates to assess how well the scores explained the outcomes (Fig. 3c and d). For the internal validation, we randomly split the 2309 patients included in the multivariable analysis into training cohort and validation cohort at a 6:4 ratio, and multivariable Cox proportional hazard models with the same algorithm were performed. C-index was not different between training cohort (0.69 [95% CI 0.65–0.72]) and validation cohort (0.68 [95% CI 0.64–0.73]).

### Distant recurrence free survival

The 3-year and 5-year DRFS rates were 94.2% and 90.2%, respectively (Fig. 2b). Univariable analysis for DRFS revealed that age, Ki-67, nuclear grade, lymphatic invasion, vascular invasion, pathological invasive tumor size, pathological number of metastatic lymph nodes, NAC, and pathological therapeutic response were significantly associated factors (Supplementary Table 2). As in the multivariable analysis for DRFS, the model including all covariates was followed by the stepwise method.

Nuclear grade (Grade 2 vs. Grade 1: HR 1.94, 95% CI 1.46–2.60, *p* < 0.001; Grade 3 vs. Grade 1: HR 1.87, 95% CI 1.33–2.64, *p* < 0.001), pathological invasive tumor size (2–5 cm vs. < 2 cm: HR 2.34, 95% CI 1.66–3.30, *p* < 0.001; ≥ 5 cm vs. < 2 cm: HR 3.05, 95% CI 2.03–4.57, *p* < 0.001), number of pathological metastatic lymph nodes (1–3 vs.0: HR 1.68, 95% CI 1.26–2.24, *p* < 0.001; ≥ 4 vs.0: HR 2.70, 95% CI 1.95–3.74, *p* < 0.001), and NAC (Yes vs. No: HR 2.67, 95% CI 2.04–3.48, *p* < 0.001) were significant prognostic factors (Table 5).

**Table 5:** Prognostic factors for DRFS in the multivariable analysis

Factor	Level	n (event)^a^	Including all covariates		Stepwise method (*p* = 0.05)	
			HR (95% CI)	*p* value^b^	HR (95% CI)	*p* value^b^
Age	20–39 years	233 (41)	Ref	(< 0.001)^c^	Ref	(< 0.001)
	40–69 years	1739 (199)	0.63 (0.44–0.91)	0.014	0.75 (0.53–1.05)	0.092
	≥ 70 years	337 (51)	1.11 (0.67–1.83)	0.695	1.46 (0.96–2.22)	0.075
Menopausal status	Premenopausal	1172 (140)	Ref	0.158		
	Postmenopausal	1137 (151)	1.23 (0.92–1.64)			
Bilateral breast cancer	No	2272 (282)	Ref	0.079		
	Yes	37 (9)	1.84 (0.93–3.64)			
ER	< 1%	15 (2)	Ref	(0.613)		
	1–9%	10 (3)	2.34 (0.38–14.31)	0.359		
	≥ 10%	1378 (177)	1.30 (0.31–5.46)	0.721		
	Unknown	906 (109)	1.11 (0.25–4.88)	0.892		
PgR	< 1%	137 (28)	Ref	(0.545)		
	≥ 1%	751 (104)	0.81 (0.51–1.28)	0.358		
	Unknown	1421 (159)	0.74 (0.43–1.28)	0.284		
HER2	0	1179 (159)	Ref	(0.993)		
	1+	817 (93)	0.99 (0.76–1.28)	0.909		
	2+	313 (39)	1.00 (0.69–1.43)	0.978		
Ki-67	< 14%	358 (34)	Ref	(0.571)		
	14–29%	299 (50)	1.27 (0.80–2.01)	0.305		
	≥ 30%	321 (65)	1.42 (0.87–2.31)	0.159		
	Unknown	1331 (142)	1.26 (0.81–1.94)	0.306		
Nuclear grade	Grade 1	958 (67)	Ref	(< 0.001)	Ref	(< 0.001)
	Grade 2	953 (157)	1.83 (1.35–2.47)	< 0.001	1.94 (1.46–2.60)	< 0.001
	Grade 3	398 (67)	1.56 (1.08–2.27)	0.019	1.87 (1.33–2.64)	< 0.001
Lymphatic invasion	No	1263 (115)	Ref	0.227		
	Yes	1046 (176)	1.19 (0.90–1.57)			
Vascular invasion	No	2035 (239)	Ref	0.232		
	Yes	274 (52)	1.23 (0.88–1.72)			
Pathological tumor size	< 2 cm	704 (41)	Ref	(< 0.001)	Ref	(< 0.001)
	2 cm to less than 5 cm	1334 (176)	2.30 (1.62–3.26)	< 0.001	2.34 (1.66–3.30)	< 0.001
	≥ 5 cm	271 (74)	2.92 (1.92–4.45)	< 0.001	3.05 (2.03–4.57)	< 0.001
Number of pathological lymph node metastases	0	1154 (81)	Ref	(< 0.001)	Ref	(< 0.001)
	1–3	837 (120)	1.58 (1.16–2.15)	0.004	1.68 (1.26–2.24)	0.001
	≥ 4	318 (90)	2.42 (1.68–3.47)	< 0.001	2.70 (1.95–3.74)	< 0.001
NAC	No	1994 (195)	Ref	< 0.001	Ref	< 0.001
	Yes	315 (96)	2.62 (1.94–3.53)		2.67 (2.04–3.48)	

*DRFS* distant recurrence-free survival, *CI* confidential interval, *HR* hazard ratio, *NAC* neoadjuvant chemotherapy, *Ref* reference

^a^Analysis was performed on the 2309 cases for which all explanatory variables were available

^b^Wald test p-value for HR

^c^Number of parentheses represents p-value for the comparison among all categories

### Overall survival

The 3-year and 5-year OS rates were 98.4% and 96.0%, respectively (Fig. 2c). Univariable analysis for OS revealed that age, menopausal status, bilateral breast cancer, Ki-67, nuclear grade, lymphatic invasion, pathological invasive tumor size, number of pathological metastatic lymph nodes, NAC, and pathological therapeutic response were significant prognostic factors (Supplementary Table 3). Significant prognostic factors for OS, as assessed by multivariable analysis using the stepwise method, were menopausal status (postmenopausal vs. premenopausal: HR 1.71, 95% CI 1.14–2.58, *p* = 0.010), bilateral breast cancer (Yes vs. No: HR 2.72, 95% CI 1.26–5.87, *p* = 0.011), nuclear grade (Grade 2 vs. Grade 1: HR 2.07, 95% CI 1.35–3.17, *p* < 0.001; Grade 3 vs. Grade 1: HR 2.17, 95% CI 1.34–3.52, *p* = 0.002), pathological invasive tumor size (2–5 cm vs. < 2 cm: HR 2.66, 95% CI 1.60–4.44, *p* < 0.001; ≥ 5 cm vs. < 2 cm: HR 3.20, 95% CI 1.78–5.77, *p* < 0.001), number of pathological metastatic lymph nodes (1–3 vs.0: HR 1.80, 95% CI 1.20–2.70, *p* = 0.004; ≥ 4 vs.0: HR 2.34, 95% CI 1.51–3.90, *p* < 0.001), and NAC (Yes vs. No: HR 3.66, 95% CI 2.55–5.26, *p* < 0.001) (Table 6).

**Table 6:** Prognostic factors for OS in the multivariable analysis

Factor	Level	n (event)^a^	Including all covariates		Stepwise method (*p* = 0.05)	
			HR (95% CI)	*p* value^b^	HR (95% CI)	*p* value^b^
Age	20–39 years	233 (17)	Ref	(< 0.001)^c^	Ref	(< 0.001)
	40–69 years	1739 (94)	0.62 (0.35–1.11)	0.109	0.66 (0.37–1.16)	0.130
	≥ 70 years	337 (38)	1.75 (0.86–3.58)	0.124	1.80 (0.89–3.65)	0.249
Menopausal status	Premenopausal	1172 (57)	Ref	0.018	Ref	0.010
	Postmenopausal	1137 (92)	1.66 (1.09–2.53)		1.71 (1.14–2.58)	
Bilateral breast cancer	No	2272 (142)	Ref	0.009	Ref	0.011
	Yes	37 (7)	2.87 (1.30–6.35)		2.72 (1.26–5.87)	
ER	< 1%	15 (2)	Ref	(0.569)		
	1–9%	10 (2)	1.85 (0.25–13.73)	0.548		
	≥ 10%	1378 (94)	0.68 (0.16–2.98)	0.610		
	Unknown	906 (51)	0.60 (0.12–2.87)	0.519		
PgR	< 1%	137 (17)	Ref	(0.751)		
	≥ 1%	751 (58)	0.87 (0.47–1.60)	0.651		
	Unknown	1421 (74)	0.75 (0.35–1.59)	0.451		
HER2	0	1179 (86)	Ref	(0.733)		
	1+	817 (46)	0.96 (0.67–1.39)	0.828		
	2+	313 (17)	0.80 (0.47–1.39)	0.431		
Ki-67	< 14%	358 (21)	Ref	(0.615)		
	14–29%	299 (23)	0.92 (0.49–1.71)	0.783		
	≥ 30%	321 (38)	1.33 (0.70–2.52)	0.387		
	Unknown	1331 (67)	1.12 (0.63–2.00)	0.698		
Nuclear grade	Grade 1	958 (30)	Ref	(0.009)	Ref	(0.002)
	Grade 2	953 (81)	2.01 (1.29–3.12)	0.002	2.07 (1.35–3.17)	< 0.001
	Grade 3	398 (38)	1.80 (1.06–3.06)	0.030	2.17 (1.34–3.52)	0.002
Lymphatic invasion	No	1263 (62)	Ref	0.400		
	Yes	1046 (87)	1.18 (0.80–1.73)			
Vascular invasion	No	2035 (122)	Ref	0.575		
	Yes	274 (27)	1.14 (0.72–1.83)			
Pathological tumor size	< 2 cm	704 (18)	Ref	(< 0.001)	Ref	(< 0.001)
	2 cm to less than 5 cm	1334 (92)	2.74 (1.63–4.61)	< 0.001	2.66 (1.60–4.44)	< 0.001
	≥ 5 cm	271 (39)	3.17 (1.72–5.85)	< 0.001	3.20 (1.78–5.77)	< 0.001
Number of pathological lymph node metastases	0	1154 (39)	Ref	(0.011)	Ref	(0.001)
	1–3	837 (67)	1.71 (1.11–2.64)	0.015	1.80 (1.20–2.70)	0.004
	≥ 4	318 (43)	2.16 (1.28–3.65)	0.004	2.34 (1.46–3.76)	< 0.001
NAC	No	1994 (92)	Ref	< 0.001	Ref	< 0.001
	Yes	315 (57)	3.59 (2.39–5.39)		3.66 (2.55–5.26)	

*OS* overall survival, *CI* confidential interval, *HR* hazard ratio, *NAC* neoadjuvant chemotherapy, *Ref* reference

^a^Analysis was performed on the 2309 cases for which all explanatory variables were available

^b^Wald test p-value for HR

^c^Number of parentheses represents p-value for the comparison among all categories

### Cumulative recurrence rates

The 3-year and 5-year cumulative recurrence rates (local and distant recurrence) were 6.8% and 11.1%, respectively (Supplementary Fig. 1a). Univariable analysis was performed to explore prognostic factors for the cumulative recurrence rate. Age, Ki-67, nuclear grade, lymphatic invasion, vascular invasion, pathological invasive tumor size, number of pathological metastatic lymph nodes, presence of NAC, and pathological therapeutic response were significant factors (Supplementary Table 4a). Significant prognostic factors for the cumulative recurrence rate in the multivariable analysis with the model including all covariates were age (40–69 vs. 20–39 years: HR 0.63, 95% CI 0.45–0.88, *p* = 0.008), nuclear grade (Grade 2 vs. Grade 1: HR 1.95, 95% CI 1.45–2.61, *p* < 0.001; Grade 3 vs. Grade 1: HR 1.83, 95% CI 1.28–2.62, *p* < 0.001), lymphatic invasion (Yes vs. No: HR 1.52, 95% CI 1.14–2.02, *p* = 0.004), pathological invasive tumor size (2–5 cm vs. < 2 cm: HR 2.06, 95% CI 1.50–2.83, *p* < 0.001; ≥ 5 cm vs. < 2 cm: HR 2.39, 95% CI 1.60–3.56, *p* < 0.001), number of pathological metastatic lymph nodes (≥ 4 vs.0: HR 1.76, 95% CI 1.23–2.51, *p* = 0.002), and NAC (Yes vs. No: HR 2.35, 95% CI 1.76–3.14, *p* < 0.001) (Supplementary Table 4b).

### Cumulative distant metastasis rates

The 3-year and 5-year cumulative distant metastasis rates were 5.3% and 8.8%, respectively (Supplementary Fig. 1b). The results of univariable analysis and multivariable analysis are shown in Supplementary Tables 5a and 5b.

### Patient background and survival outcomes in the patient population meeting the eligibility criteria for the monarchE trial

We examined the clinicopathological characteristics and IDFS rate of subgroups that met the Cohort 1 and Cohort 2 criteria of the monarchE trial [3]. Histological grade was substituted for nuclear grade and defined as follows for Cohort 1 and Cohort 2: Cohort 1, patients with either (1) ≥ 4 lymph nodes or (2) 1–3 lymph nodes and tumor size ≥ 5 cm or nuclear Grade 3; Cohort 2, patients with 1–3 lymph nodes, tumor size < 5 cm, nuclear Grade 1 or 2, and Ki-67 ≥ 20%. Clinicopathological characteristics and treatment details for the subgroups are shown in Supplementary Tables 6–8. Kaplan–Meier curves for IDFS in the same population are shown in Supplementary Figs. 1a-1c. The 3-year and 5-year IDFS rates were 85.6% and 77.2%, respectively, in the present study population that corresponded to the intention to treat population of the monarchE trial.

## Discussion

We investigated the clinicopathological features associated with early recurrence within three years after surgery in patients with HR+/HER2− breast cancer.

It is noteworthy that younger age and vascular invasion were identified as independent risk factors for early recurrence in the present study. The inclusion criteria of the monarchE trial did not contain these factors [3]. In the present study, those aged 20–39 years had 3-year IDFS rate of 89.0%. Previous studies have confirmed that adolescent and young adult women present with biologically aggressive disease, with higher rates of HR-negative, higher grade breast tumors, and more lymphovascular invasion than elderly patients, which is linked to poor prognosis [9–11]. Young Asian patients aged < 40 years with HR+/HER2− subtypes are more likely to have worse survival outcomes than middle-aged patients (40–49 years) [12]. Moreover, the luminal B subtype is predominant in young patients and is associated with a poor prognosis [13–15]. In addition, genomic factors were linked to poor prognosis in young patients with breast cancer in previous studies [16]. Although these previous studies did not focus solely on early recurrence, they support the results of the present study.

The univariable analysis for IDFS showed that patients aged ≥ 70 years had a worse prognosis than those aged 40–69 years (Supplementary Table 1a). In contrast, the 3-year cumulative recurrence rate was not markedly different between patients aged 40–69 years and those aged ≥ 70 years (Supplementary Table 4a). Although cause of death was not investigated in the present study, non-breast cancer deaths or second primary non-breast cancer might have contributed to the high IDFS events by 3 years in elderly patients, as suggested in previous studies [17, 18].

Lymphovascular invasion is a prognostic factor for 10-year recurrence and an independent predictor of metastasis, particularly for patients with node-negative breast cancer [19, 20]. Vascular invasion, but not lymphatic invasion, was an independent risk factor for recurrence in previous studies [21, 22]. Consistent with this, vascular invasion was identified as an independent risk factor for early recurrence in the present study. The presence of vascular invasion may be an indicator of high biological invasiveness and better represent systemic disease than lymphatic invasion and may be associated with early recurrence [21].

Consistent with previous studies [5, 6, 23], large tumor size, a high number of positive lymph nodes, and Grade 3 disease were found to be risk factors for early recurrence. These factors were included in the eligibility criteria for monarchE. In the present study, both Grade 2 disease and Grade 3 disease had a poorer prognosis than Grade 1 disease. In the nomogram, Grade 2 disease appeared to have a slightly poorer prognosis than Grade 3 (Fig. 3a). However, we posit that there were no clinically meaningful differences between the two. Because central pathological diagnosis was not performed, pre-analytical variables and analytical differences may explain the lack of a clear difference between Grade 2 and Grade 3 disease. Nuclear grade, pathological invasive tumor size, number of metastatic pathological lymph nodes, and the presence of NAC were common prognostic factors in the multivariable analysis for both DRFS and OS. Although low ER expression was not identified as a risk factor for early recurrence, previous studies linked low ER expression to a high risk [5, 6]. As the number of patients with tumors having low ER expression was small in the present study, further investigation is needed. In addition, Ki-67 was not identified as an early recurrence risk factor, likely due to measurements not being central and a high number of missing values.

NAC was an independent prognostic factor associated with early recurrence in this cohort. Patients who received NAC had worse survival suggesting that NAC is being implemented in patients at a high risk of early recurrence. For instance, patients aged 20–39 years with nuclear Grade 2, vascular invasion, tumor size 2–5 cm, and 1–3 positive lymph nodes who have undergone NAC have an estimated 3-year IDFS rate of < 75%, according to the nomogram. This rate is lower than the 83.4% rate observed in patients of the endocrine-therapy alone arm of the monarchE trial [3]. Further studies are needed to evaluate the efficacy of abemaciclib as an additional postoperative treatment for patients with such high-risk disease.

As previously mentioned, NAC is often administered to patients whom physicians consider to be at high risk of recurrence. However, there is a lack of consensus regarding the indication of NAC for HR+/HER2− breast cancer, leading to potential selection bias among physicians and institutions. Therefore, we conducted a multivariable analysis for IDFS to compare the overall cohort, including patients who received NAC, with the cohort of patients who did not receive NAC. As a result, the significant factors identified in the cohort of patients who did not receive NAC were generally similar to those in the overall cohort. In particular, nuclear grade, pathological invasive tumor size, and the number of pathological lymph node metastases were considered significant risk factors for recurrence in all cohorts. Lymphatic invasion, along with vascular invasion, has also been reported in previous studies as an important risk factor [24, 25].

This study has several potential limitations. First, there are missing values due to the retrospective nature of data collection. Second, our nomogram was generated based on the data from five institutions in Japan and has not been validated for generalizability to other cohorts. Third, collected data did not include information on the *BRCA1/2* gene or results of the multigene assay due to limited number of testing because *BRCA* mutation testing and the multigene assay were not covered by insurance in Japan during the study period. The question of whether genomic risk factors contribute to early recurrence remains a topic for future investigation. As a next step, we will compare the risk of recurrence and differences in molecular subtypes between patients with early recurrence and those without recurrence using MammaPrint/BluePrint (UMIN 000050930).

## Conclusion

We identified younger age, higher nuclear grade, presence of vascular invasion, larger pathological invasive tumor size, larger number of pathologic lymph node metastases as risk factors for early postoperative recurrence in HR+/HER2− breast cancer. In addition, the patients selected for NAC by the attending physician were at a high risk of early recurrence even receiving NAC. More studies are warranted to assess the efficacy of abemaciclib in patients with early recurrence risk factors highlighted in this study. Additional research, including external validation, is needed and would improve the accuracy of prognosis prediction in combination with results from multigene assays.

## Supplementary Information

Below is the link to the electronic supplementary material.

Supplementary file1 (PDF 81 KB)

Supplementary file2 Supplementary Fig. 1a) Cumulative incidence curve for cumulative recurrence rates (local and distant recurrence), b) Cumulative incidence curve for cumulative distant metastasis rates. Supplementary Fig. 2a) IDFS curves for subgroups that correspond to the intent-to-treat population of the monarchE trial in the present study, b) IDFS curves for subgroups that correspond to the cohort1 population of the monarchE trial in the present study, c) IDFS curves for subgroups that correspond to the cohort2 population of the monarchE trial in the present study (PDF 156 KB)

Supplementary file3 (DOCX 156 KB)

## Data Availability

The datasets generated during and/or analyzed during this current study are available from the corresponding author on reasonable request.
