# Supplementary material for: Risk factors for early recurrence in patients with hormone receptor-positive, HER2-negative breast cancer: a retrospective cohort study in Japan (WJOG15721B)
Source: Breast Cancer. 2025 Apr 10;32(4):757–72. doi: 10.1007/s12282-025-01700-y (PMC12174269; doi:10.1007/s12282-025-01700-y)
Supplement: Supplementary file 1 — Supplementary file1 (PDF 81 KB) [file 12282_2025_1700_MOESM1_ESM.pdf]

Supplementary appendix

WJOG15721B RealisE Principal Investigators	
**PI Name**	**Affiliation**
Naoki Hayashi/Atsushi Yoshida	St. Luke's International Hospital
Toshimi Takano	Cancer Institute Hospital of the Japanese Foundation for Cancer Research
Rurina Watanuki	National Cancer Center Hospital East
Hidenori Kamio	Tokyo Metropolitan Cancer and Infectious Diseases Center Komagome Hospital
Atsushi Fushimi	The Jikei University School of Medicine
