# Supplementary material for: Risk factors for early recurrence in patients with hormone receptor-positive, HER2-negative breast cancer: a retrospective cohort study in Japan (WJOG15721B)
Source: Breast Cancer. 2025 Apr 10;32(4):757–72. doi: 10.1007/s12282-025-01700-y (PMC12174269; doi:10.1007/s12282-025-01700-y)
Supplement: Supplementary file 2 — Supplementary file2 Supplementary Fig. 1a) Cumulative incidence curve for cumulative recurrence rates (local and distant recurrence), b) Cumulative incidence curve for cumulative distant metastasis rates. Supplementary Fig. 2a) IDFS curves for subgroups that correspond to the intent-to-treat population of the monarchE trial in the present study, b) IDFS curves for subgroups that correspond to the cohort1 population of the monarchE trial in the present study, c) IDFS curves for subgroups that correspond to the cohort2 population of the monarchE trial in the present study (PDF 156 KB) [file 12282_2025_1700_MOESM2_ESM.pdf]

Supplementary Fig. 1a

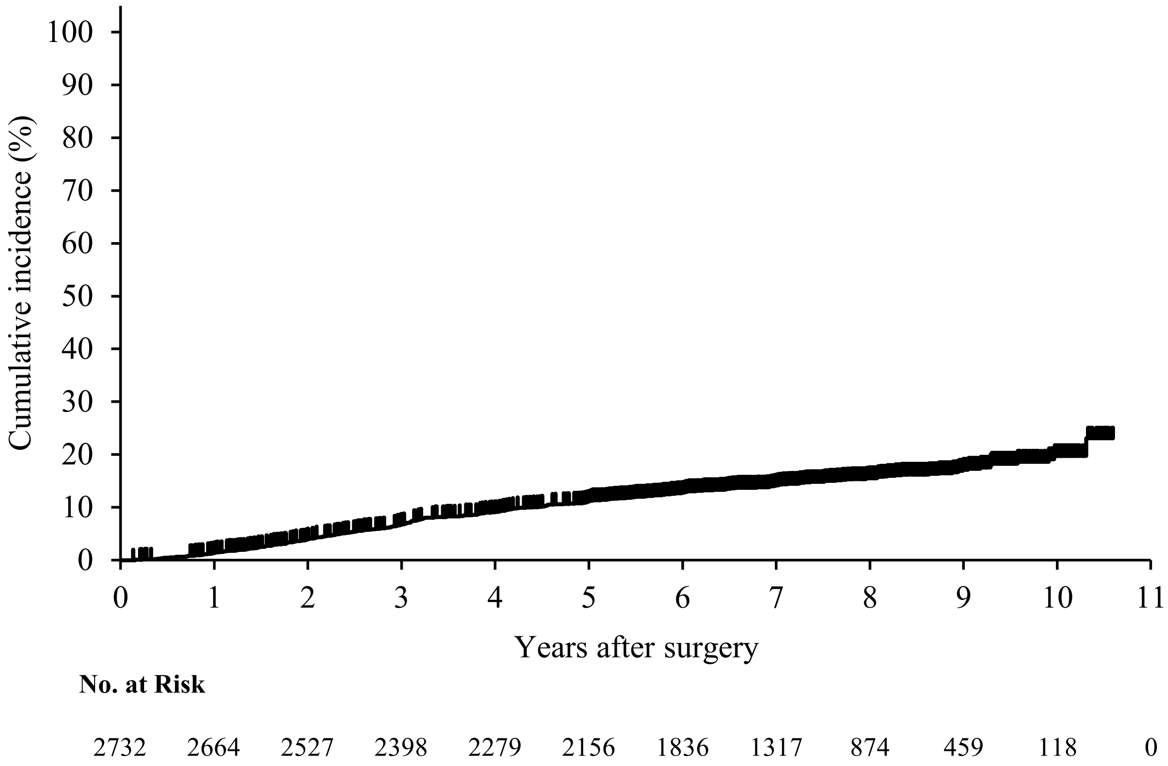

Event	Competitive risk event	1-year recurrence rate （95%CI）	2-year recurrence rate （95%CI）	3-year recurrence rate （95%CI）	4-year recurrence rate （95%CI）	5-year recurrence rate （95%CI）
395	64	1.4% （1.0 – 1.9 %）	3.9% （3.3 – 4.7 %）	6.7% （5.8 – 7.7 %）	9.1% （8.0 – 10.2 %）	11.1% （10.0 – 12.4 %）

Supplementary Fig. 1b

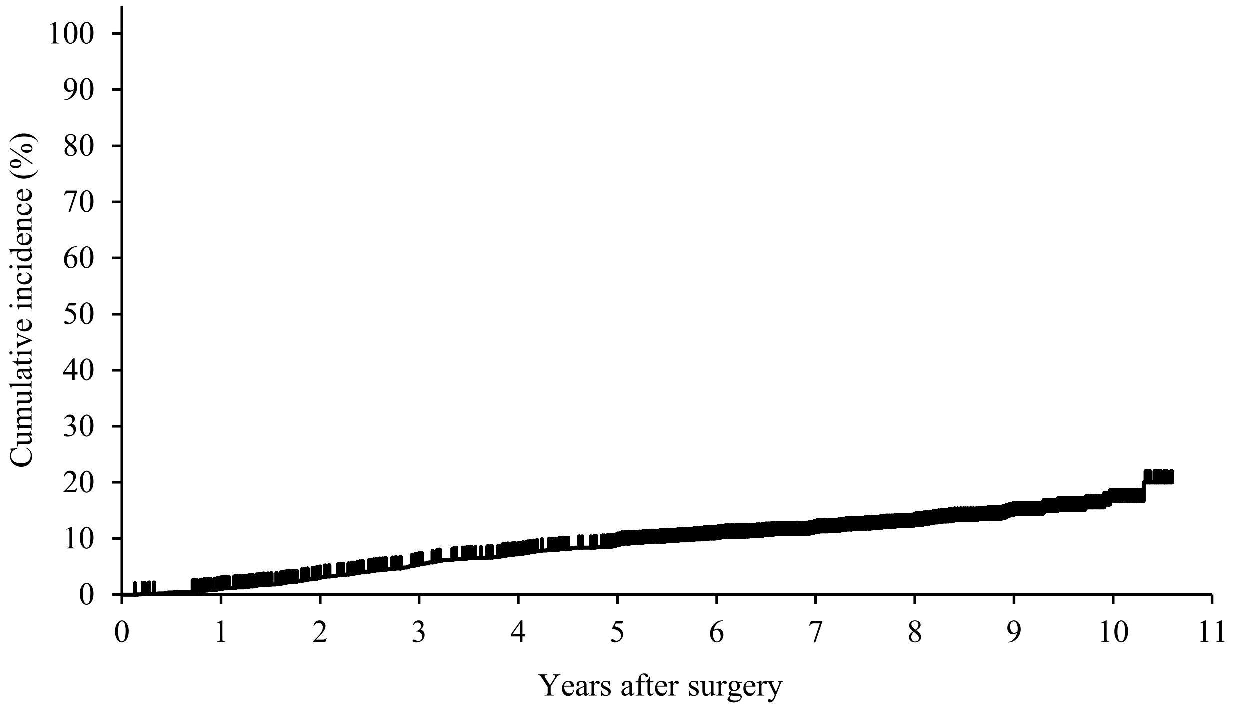

No. at Risk

2732	2667	2534	2403	2285	2162	1842	1322	879	460	118	0

Event	Competitive risk event	1-year distant metastasis rate （95%CI）	2-year distant metastasis rate （95%CI）	3-year distant metastasis rate （95%CI）	4-year distant metastasis rate （95%CI）	5-year distant metastasis rate （95%CI）
317	142	1.0% （0.7 – 1.5 %）	3.0% （2.4 – 3.7 %）	5.3% （4.5 – 6.2 %）	7.1% （6.2 – 8.2 %）	8.8% （7.8 – 9.9 %）

Supplementary Fig. 2a

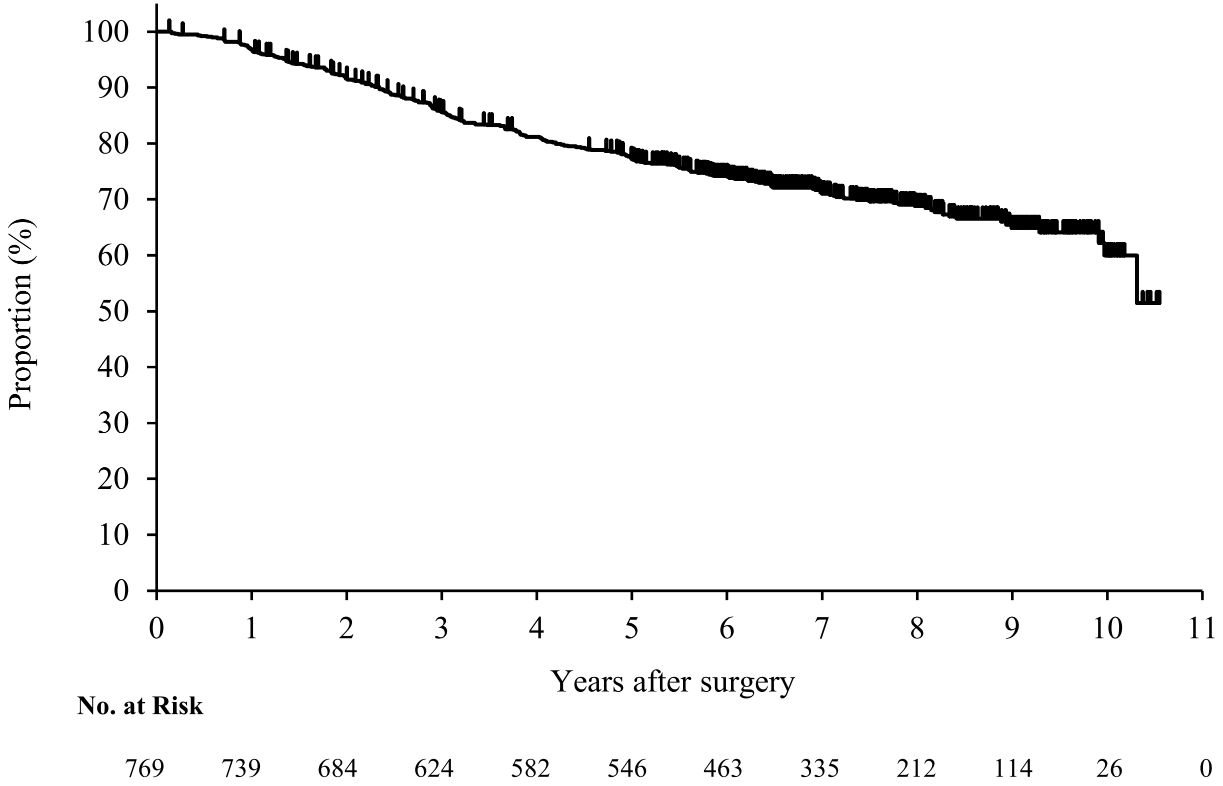

Event	1-year IDFS (95%CI)	2-year IDFS (95%CI)	3-year IDFS (95%CI)	4-year IDFS (95%CI)	5-year IDFS (95%CI)
228	96.7% (95.2 – 97.8 %)	91.6% (89.4 – 93.3 %)	85.6% (82.9 – 87.9 %)	81.2% (78.2 – 83.8 %)	77.2% (74.0 – 80.1 %)

Supplementary Fig. 2b

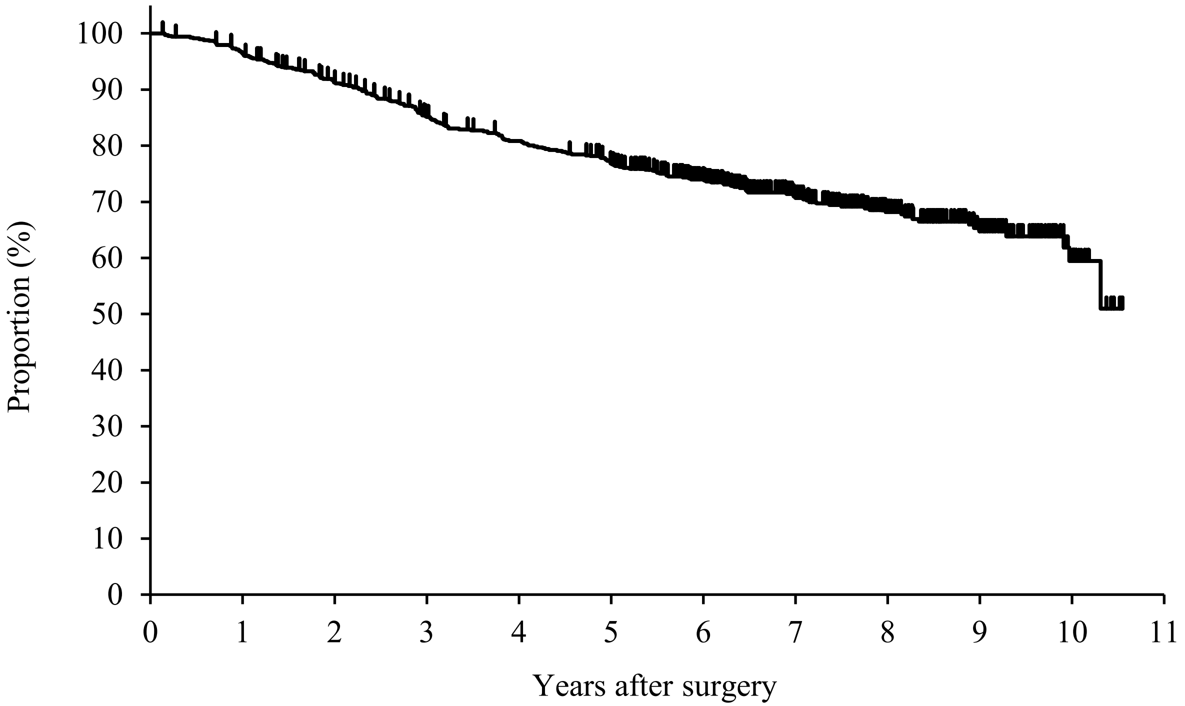

No. at Risk

673	645	597	543	510	477	405	295	187	105	24	0

Event	1-year IDFS (95%CI)	2-year IDFS (95%CI)	3-year IDFS (95%CI)	4-year IDFS (95%CI)	5-year IDFS (95%CI)
203	96.4% (94.7 – 97.6 %)	91.3% (88.9 – 93.2 %)	85.1% (82.1 – 87.6 %)	80.8% (77.6 – 83.6 %)	76.8% (73.4 – 79.9 %)

Supplementary Fig. 2c

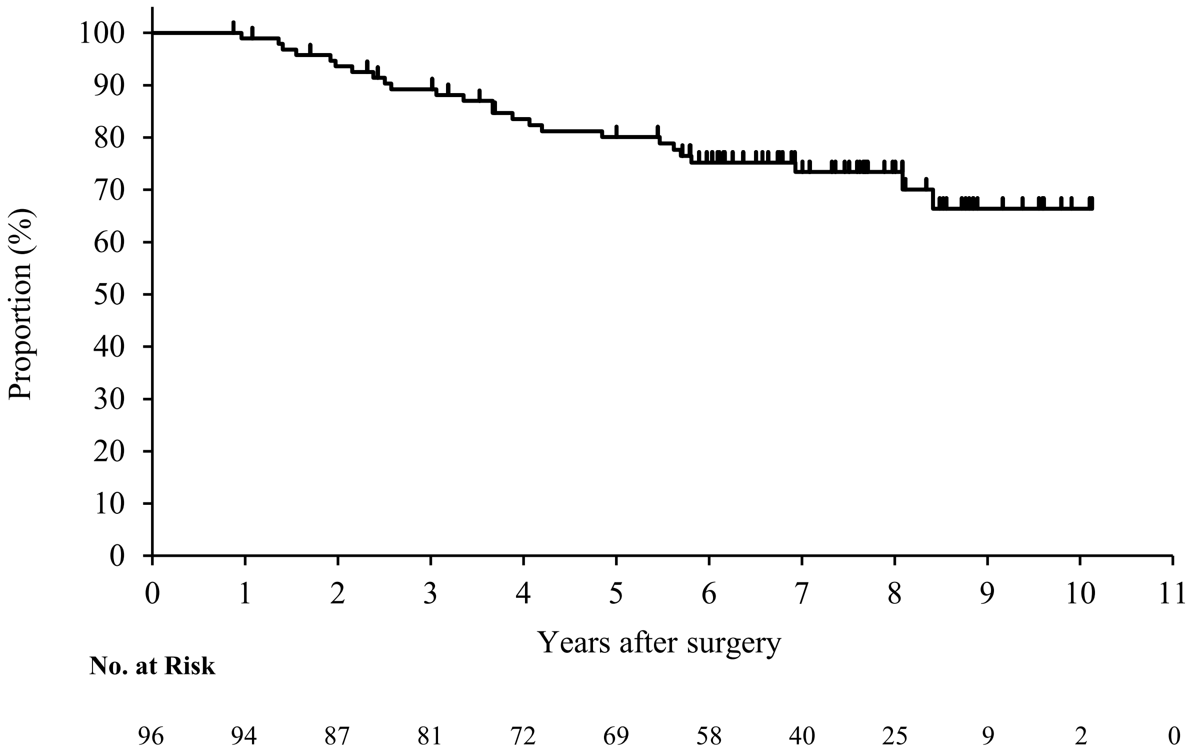

Event	1-year IDFS （95%CI）	2-year IDFS （95%CI）	3-year IDFS （95%CI）	4-year IDFS （95%CI）	5-year IDFS （95%CI）
25	98.9% （92.8 – 99.9 %）	93.6% （86.3 – 97.1 %）	89.2% （80.9 – 94.1 %）	83.5% （74.2 – 89.7 %）	80.1% （70.2 – 87.0 %）
