# Supplementary material for: Risk factors for early recurrence in patients with hormone receptor-positive, HER2-negative breast cancer: a retrospective cohort study in Japan (WJOG15721B)
Source: Breast Cancer. 2025 Apr 10;32(4):757–72. doi: 10.1007/s12282-025-01700-y (PMC12174269; doi:10.1007/s12282-025-01700-y)
Supplement: Supplementary file 3 — Supplementary file3 (DOCX 156 KB) [file 12282_2025_1700_MOESM3_ESM.docx]

**Supplementary Table 1a** Prognostic factors for IDFS in the univariable analysis

| Factor | Level | n (event) | 3-year IDFS rate (95%CI) | HR (95% CI) | *p* value^*1^ |
| --- | --- | --- | --- | --- | --- |
| Age | 20-39 years | 274 (68) | 85.6% (80.7–89.3 %) | Ref | (<0.001)^*2^ |
|  | 40-69 years | 2094 (335) | 93.1% (91.9–94.1 %) | 0.59 (0.46 – 0.77) | <0.001 |
|  | ≥70 years | 364 (76) | 91.6% (88.2–94.1 %) | 0.96 (0.69 – 1.33) | 0.803 |
| Menopausal status | Premenopausal | 1395 (238) | 91.5% (89.9–92.9 %) | Ref | 0.183 |
|  | Postmenopausal | 1311 (240) | 92.8% (91.2–94.1 %) | 1.13 (0.94 – 1.35) |  |
| Bilateral breast cancer | No | 2690 (469) | 92.3% (91.2–93.3 %) | Ref | 0.285 |
|  | Yes | 42 (10) | 83.3% (68.2–91.7 %) | 1.41 (0.75 – 2.63) |  |
| ER | <1% | 15 (3) | 93.3% (61.3–99.0 %) | Ref | (0.551) |
|  | 1-9% | 18 (5) | 71.1% (43.8–86.9 %) | 1.90 (0.45 – 7.93) | 0.381 |
|  | ≥10% | 1722 (297) | 92.6% (91.2–93.8 %) | 0.99 (0.32 – 3.10) | 0.992 |
|  | Unknown | 977 (174) | 91.7% (89.8–93.3 %) | 1.03 (0.33 – 3.22) | 0.964 |
| PgR | <1% | 152 (33) | 87.8% (81.4–92.1 %) | Ref | (0.019) |
|  | ≥1% | 825 (160) | 92.0% (89.9–93.7 %) | 0.87 (0.60 – 1.27) | 0.480 |
|  | Unknown | 1755 (286) | 92.6% (91.2–93.7 %) | 0.69 (0.48 – 0.99) | 0.046 |
| HER2 | 0 | 1386 (253) | 92.0% (90.4–93.3 %) | Ref | (0.729) |
|  | 1+ | 1008 (169) | 92.3% (90.5–93.8 %) | 0.92 (0.76 – 1.12) | 0.428 |
|  | 2+ | 338 (57) | 92.3% (88.8–94.7 %) | 0.98 (0.73 – 1.30) | 0.881 |
| Ki-67 | <14% | 371 (56) | 93.6% (90.5–95.7 %) | Ref | (<0.001) |
|  | 14-29% | 315 (64) | 91.4% (87.6–94.1 %) | 1.42 (0.99 – 2.03) | 0.057 |
|  | ≥30% | 367 (94) | 86.0% (81.9–89.2 %) | 1.90 (1.37 – 2.65) | 0.001 |
|  | Unknown | 1679 (265) | 93.3% (92.0–94.4 %) | 1.04 (0.78 – 1.38) | 0.813 |
| Nuclear grade | Grade 1 | 1041 (120) | 94.9% (93.4–96.1 %) | Ref | (<0.001) |
|  | Grade 2 | 1039 (219) | 91.5% (89.6–93.1 %) | 1.84 (1.47 – 2.30) | <0.001 |
|  | Grade 3 | 456 (97) | 88.2% (84.8–90.8 %) | 1.87 (1.43 – 2.45) | <0.001 |
| Histological grade | Grade 1 | 149 (27) | 91.9% (86.1–95.3 %) | Ref | (0.267) |
|  | Grade 2 | 218 (55) | 89.8% (84.9–93.2 %) | 1.44 (0.91 – 2.27) | 0.1245 |
|  | Grade 3 | 69 (20) | 86.8% (76.2–92.9 %) | 1.46 (0.81 – 2.63) | 0.208 |
| Lymphatic invasion | No | 1545 (218) | 94.1% (92.8–95.2 %) | Ref | <0.001 |
|  | Yes | 1166 (259) | 89.4% (87.5–91.1 %) | 1.67 (1.40 – 2.00) |  |
| Vascular invasion | No | 2427 (409) | 92.3% (91.1–93.3 %) | Ref | 0.003 |
|  | Yes | 284 (68) | 90.8% (86.7–93.7 %) | 1.47 (1.14 – 1.90) |  |
| Pathological tumor size | <2 cm | 765 (85) | 95.8% (94.1–97.0 %) | Ref | (<0.001) |
|  | 2 cm to less than 5 　　cm | 1423 (255) | 92.3% (90.7–93.6 %) | 1.68 (1.31 – 2.15) | <0.001 |
|  | ≥5 cm | 286 (87) | 83.4% (78.5–87.3 %) | 3.14 (2.33 – 4.23) | <0.001 |
| Number of pathological lymph node metastases | 0 | 1317 (164) | 94.5% (93.1–95.6 %) | Ref | (<0.001) |
|  | 1-3 | 995 (178) | 92.8% (91.0–94.2 %) | 1.44 (1.16 – 1.78) | 0.001 |
|  | ≥4 | 404 (131) | 84.1% (80.1–87.3 %) | 2.88 (2.29 – 3.62) | <0.001 |
| NAC | No | 2103 (306) | 93.8% (92.7–94.8 %) | Ref | <0.001 |
|  | Yes | 628 (172) | 86.5% (83.6–89.0 %) | 2.06 (1.71 – 2.48) |  |
| Pathological therapeutic response | Grade 0 | 42 (16) | 68.5% (52.0–80.4 %) | Ref | (0.016) |
|  | Grade 1 | 454 (124) | 87.7% (84.2–90.4 %) | 0.60 (0.35 – 1.00) | 0.051 |
|  | Grade 2 | 154 (42) | 86.2% (79.6–90.8 %) | 0.60 (0.33 – 1.06) | 0.078 |
|  | Grade 3 | 50 (5) | 97.9% (86.1–99.7 %) | 0.20 (0.07 – 0.54) | 0.002 |

*1 Wald test p-value for HR

*2 Number of parentheses represents p-value for the comparison among all categories.

Abbreviations: IDFS, invasive disease-free survival; CI, confidential interval; HR, hazard ratio; NAC, neoadjuvant chemotherapy

**Supplementary Table 1b** Prognostic factors for IDFS in patients who did not receive neoadjuvant chemotherapy by univariable analysis

| Factor | Level | n (event) | 3-year IDFS rate (95%CI) | HR (95% CI) | *p* value^*1^ |
| --- | --- | --- | --- | --- | --- |
| Age | 20-39 years | 183 (34) | 89.7% (84.1–93.4 %) | Ref | (<0.001)^*2^ |
|  | 40-69 years | 1577 (206) | 94.7% (93.3–95.6 %) | 0.65 (0.45 – 0.94) | 0.020 |
|  | ≥70 years | 343 (66) | 92.6% (89.1–95.0 %) | 1.24 (0.82 – 1.87) | 0.313 |
| Menopausal status | Premenopausal | 995 (133) | 93.3% (91.5–94.7 %) | Ref | 0.043 |
|  | Postmenopausal | 1089 (172) | 94.3% (92.7–95.5 %) | 1.26 (1.01 – 1.58) |  |
| Bilateral breast cancer | No | 2072 (299) | 94.0% (92.9–94.9 %) | Ref | 0.192 |
|  | Yes | 31 (7) | 83.9% (65.5–92.9 %) | 1.65 (0.78 – 3.48) |  |
| ER | <1% | 6 (0) | 100% (100–100 %) | Ref | - |
|  | 1-9% | 9 (1) | 88.9% (43.3–98.4 %) | NE | - |
|  | ≥10% | 1306 (191) | 93.9% (92.5–95.1 %) | NE | - |
|  | Unknown | 782 (114) | 93.7% (91.7–95.2 %) | NE | - |
| PgR | <1% | 113 (20) | 89.0% (81.5–93.6 %) | Ref | (0.136) |
|  | ≥1% | 638 (101) | 93.7% (91.4–95.3 %) | 0.87 (0.54 – 1.41) | 0.581 |
|  | Unknown | 1352 (185) | 94.3% (92.9–95.4 %) | 0.71 (0.45 – 1.13) | 0.152 |
| HER2 | 0 | 1062 (161) | 94.1% (92.5–95.4 %) | Ref | (0.582) |
|  | 1+ | 769 (113) | 93.4% (91.3–95.0 %) | 0.99 (0.78 – 1.26) | 0.922 |
|  | 2+ | 272 (32) | 93.9% (90.2–96.2 %) | 0.82 (0.56 – 1.20) | 0.305 |
| Ki-67 | <14% | 342 (40) | 95.2% (92.2–97.0 %) | Ref | (0.052) |
|  | 14-29% | 247 (41) | 92.4% (88.2–95.1 %) | 1.52 (0.98 – 2.35) | 0.061 |
|  | ≥30% | 188 (35) | 91.2% (86.0–94.5 %) | 1.77 (1.12 – 2.78) | 0.014 |
|  | Unknown | 1326 (190) | 94.1% (92.7–95.3 %) | 1.22 (0.87 – 1.71) | 0.258 |
| Nuclear grade | Grade 1 | 915 (91) | 94.9% (93.2–96.2 %) | Ref | (<0.001) |
|  | Grade 2 | 803 (142) | 93.7% (91.8–95.2 %) | 1.75 (1.35 – 2.28) | <0.001 |
|  | Grade 3 | 319 (62) | 91.1% (87.3–93.7 %) | 1.91 (1.38 – 2.64) | <0.001 |
| Histological grade | Grade 1 | 109 (10) | 97.2% (91.6–99.1 %) | Ref | (0.064) |
|  | Grade 2 | 188 (38) | 92.4% (87.6–95.5 %) | 2.29 (1.14 – 4.59) | 0.020 |
|  | Grade 3 | 38 (6) | 89.3% (74.0–95.9 %) | 1.73 (0.63 – 4.75) | 0.292 |
| Lymphatic invasion | No | 1201 (140) | 95.3% (93.9–96.4 %) | Ref | <0.001 |
|  | Yes | 898 (165) | 91.8% (89.8–93.4 %) | 1.64 (1.31 – 2.05) |  |
| Vascular invasion | No | 1846 (253) | 93.9% (92.7–94.9 %) | Ref | 0.004 |
|  | Yes | 252 (52) | 93.0% (89.0–95.6 %) | 1.54 (1.15 – 2.08) |  |
| Pathological tumor size | <2 cm | 633 (61) | 96.1% (94.2–97.4 %) | Ref | (<0.001) |
|  | 2 cm to less than 5 　　cm | 1272 (203) | 93.5% (92.0–94.8 %) | 1.71 (1.29 – 2.278) | <0.001 |
|  | ≥5 cm | 188 (40) | 88.5% (82.9–92.4 %) | 2.36 (1.58 – 3.52) | <0.001 |
| Number of pathological lymph node metastases | 0 | 1113 (134) | 94.5% (93.0–95.7 %) | Ref | (<0.001) |
|  | 1-3 | 732 (102) | 95.1% (93.2–96.5 %) | 1.12 (0.87 – 1.45) | 0.389 |
|  | ≥4 | 243 (65) | 88.1% (83.3–91.7 %) | 2.31 (1.72 – 3.10) | <0.001 |

*1 Wald test p-value for HR

*2 Number of parentheses represents p-value for the comparison among all categories.

Abbreviations: IDFS, invasive disease-free survival; CI, confidential interval; HR, hazard ratio

**Supplementary Table 1c** Prognostic factors for IDFS in patients who did not receive neoadjuvant chemotherapy by multivariable analysis

| Factor | Level | n (event)^*1^ | Including all covariates | | Stepwise method (*p*=0.05) | |
| --- | --- | --- | --- | --- | --- | --- |
|  |  |  | HR (95% CI)^*2^ | *p* value^*3^ | HR (95% CI) | *p* value^*3^ |
| Age | 20-39 years | 182 (34) | Ref | (<0.001) | Ref | (<0.001) |
|  | 40-69 years | 1488 (195) | 0.64 (0.43 – 0.95) | 0.028 | 0.18 (0.50 – 1.04) | 0.077 |
|  | ≥70 years | 324 (61) | 1.10 (0.66 – 1.84) | 0.718 | 1.40 (0.92 – 2.15) | 0.120 |
| Menopausal status | Premenopausal | 969 (129) | Ref | 0.293 |  |  |
|  | Postmenopausal | 1025 (161) | 1.17 (0.87 – 1.56) |  |  |  |
| Bilateral breast cancer | No | 1965 (283) | Ref | 0.089 |  |  |
|  | Yes | 29 (7) | 1.91 (0.91 – 4.02) |  |  |  |
| ER | <1% | 6 (0) | Ref | (0.427) |  |  |
|  | 1-9% | 6 (0) | 1.29 (0.02 – 69.05) | 0.900 |  |  |
|  | ≥10% | 1225 (179) | 2.60 (0.15 – 45.47) | 0.513 |  |  |
|  | Unknown | 757 (111) | 3.33 (0.19 – 59.19) | 0.414 |  |  |
| PgR | <1% | 110 (20) | Ref | (0.104) |  |  |
|  | ≥1% | 616 (98) | 0.84 (0.52 – 1.37) | 0.491 |  |  |
|  | Unknown | 1268 (172) | 0.54 (0.30 – 0.10) | 0.049 |  |  |
| HER2 | 0 | 1006 (155) | Ref | (0.387) |  |  |
|  | 1+ | 725 (104) | 1.02 (0.79 – 1.31) | 0.885 |  |  |
|  | 2+ | 263 (31) | 0.77 (0.52 – 1.15) | 0.198 |  |  |
| Ki-67 | <14% | 332 (39) | Ref | (0.393) |  |  |
|  | 14-29% | 240 (41) | 1.39 (0.88 – 2.20) | 0.154 |  |  |
|  | ≥30% | 175 (33) | 1.47 (0.89 – 2.41) | 0.135 |  |  |
|  | Unknown | 1247 (177) | 1.41 (0.86 – 2.33) | 0.174 |  |  |
| Nuclear grade | Grade 1 | 895 (89) | Ref | (0.004) | Ref | (0.002) |
|  | Grade 2 | 792 (141) | 1.61 (1.21 – 2.13) | 0.001 | 1.61 (1.23 – 2.10) | 0.001 |
|  | Grade 3 | 307 (60) | 1.44 (1.00 – 2.07) | 0.050 | 1.59 (1.14 – 2.23) | 0.007 |
| Lymphatic invasion | No | 1746 (238) | Ref | 0.053 | Ref | 0.047 |
|  | Yes | 248 (52) | 1.22 (0.88 – 1.70) |  | 1.32 (1.00 – 1.74) |  |
| Vascular invasion | No | 1746 (238) | Ref | 0.238 |  |  |
|  | Yes | 248 (52) | 1.22 (0.88 – 1.70) |  |  |  |
| Pathological tumor size | <2 cm | 601 (55) | Ref | (0.011) | Ref | (0.001) |
|  | 2 cm to less than 5 cm | 1209 (195) | 1.55 (1.14 – 2.10) | 0.005 | 1.66 (1.23 – 2.25) | 0.001 |
|  | ≥5cm | 184 (40) | 1.75 (1.13 – 2.71) | 0.012 | 2.01 (1.31 – 3.06) | 0.001 |
| Number of pathological lymph node metastases | 0 | 1056 (127) | Ref | (0.003) | Ref | (0.002) |
|  | 1-3 | 702 (100) | 0.97 (0.73 – 1.30) | 0.844 | 0.95 (0.72 – 1.27) | 0.750 |
|  | ≥4 | 236 (63) | 1.66 (1.17 – 2.36) | 0.005 | 1.66 (1.18 – 2.35) | 0.004 |

*1 Analysis was performed on the 1994 cases for which all explanatory variables were available.

*2 Using Firth’s method

*3 Wald test p-value for HR

*4 Number of parentheses represents p-value for the comparison among all categories.

Abbreviations: IDFS, invasive disease-free survival; CI, confidential interval; HR, hazard ratio

**Supplementary Table 2** Prognostic factors for DRFS in the univariable analysis

| Factor | Level | n (event） | 3-year DRFS rate (95%CI) | HR (95% CI) | *p* value^*1^ |
| --- | --- | --- | --- | --- | --- |
| Age | 20-39 years | 274 (53) | 89.0% (84.5–92.2 %) | Ref | (<0.001)^*2^ |
|  | 40-69 yeras | 2094 (250) | 95.0% (93.9–95.8 %) | 0.58 (0.43 – 0.78) | <0.001 |
|  | ≥70 years | 364 (59) | 93.6% (90.5–95.8 %) | 0.98 (0.68 – 1.42) | 0.920 |
| Menopausal status | Premenopausal | 1395 (176) | 93.9% (92.5–95.1 %) | Ref | 0.095 |
|  | Postmenopausal | 1311 (185) | 94.5% (93.1–95.6 %) | 1.19 (0.97 – 1.47) |  |
| Bilateral breast cancer | No | 2690 (353) | 94.3% (93.4–95.2 %) | Ref | 0.118 |
|  | Yes | 42 (9) | 85.7% (70.9–93.3 %) | 1.70 (0.88 – 3.29) |  |
| ER | <1% | 15 (2) | 93.3% (61.3–99.0 %) | Ref | (0.133) |
|  | 1-9% | 18 (5) | 71.1% (43.8–86.9 %) | 2.92 (0.57 – 15.04) | 0.201 |
|  | ≥10% | 1722 (235) | 94.3% (93.0–95.3 %) | 1.18 (0.29 – 4.75) | 0.814 |
|  | Unknown | 977 (120) | 94.5% (92.9–95.8 %) | 1.05 (0.26 – 4.24) | 0.949 |
| PgR | <1% | 152 (28) | 89.8% (83.6–93.7 %) | Ref | (0.031) |
|  | ≥1% | 825 (117) | 94.1% (92.3–95.6 %) | 0.77 (0.51 – 1.16) | 0.207 |
|  | Unknown | 1755 (217) | 94.6% (93.4–95.6 %) | 0.63 (0.43 – 0.93) | 0.021 |
| HER2 | 0 | 1386 (193) | 93.9% (92.5–95.0 %) | Ref | (0.591) |
|  | 1+ | 1008 (125) | 94.8% (93.2–96.0 %) | 0.89 (0.71 – 1.12) | 0.318 |
|  | 2+ | 338 (44) | 93.8% (90.6–96.0 %) | 0.99 (0.72 – 1.38) | 0.967 |
| Ki-67 | <14% | 371 (35) | 95.8% (93.2–97.5 %) | Ref | (<0.001) |
|  | 14-29% | 315 (50) | 94.3% (91.1–96.4 %) | 1.77 (1.15 – 2.72) | 0.010 |
|  | ≥30% | 367 (73) | 87.9% (84.0–90.9 %) | 2.35 (1.57 – 3.52) | <0.001 |
|  | Unknown | 1679 (204) | 95.2% (94.0–96.1 %) | 1.28 (0.90 – 1.84) | 0.171 |
| Nuclear grade | Grade 1 | 1041 (80) | 97.1% (95.9–98.0 %) | Ref | (<0.001) |
|  | Grade 2 | 1039 (169) | 93.1% (91.3–94.5 %) | 2.11 (1.62 – 2.76) | <0.001 |
|  | Grade 3 | 456 (74) | 91.3% (88.2–93.5 %) | 2.11 (1.54 – 2.90) | <0.001 |
| Histological grade | Grade 1 | 149 (19) | 93.9% (88.6–96.8 %) | Ref | (0.232) |
|  | Grade 2 | 218 (41) | 92.1% (87.6–95.0 %) | 1.49 (0.86 – 2.56) | 0.152 |
|  | Grade 3 | 69 (17) | 86.8% (76.2–92.9 %) | 1.71 (0.88 – 3.34) | 0.115 |
| Lymphatic invasion | No | 1545 (158) | 96.0% (94.9–96.9 %) | Ref | <0.001 |
|  | Yes | 1166 (202) | 91.7% (90.0–93.2 %) | 1.79 (1.45 – 2.20) |  |
| Vascular invasion | No | 2427 (307) | 94.3% (93.3–95.2 %) | Ref | 0.006 |
|  | Yes | 284 (53) | 93.9% (89.7–95.8 %) | 1.51 (1.13 – 2.02) |  |
| Pathological tumor size | <2 cm | 765 (48) | 98.0% (96.7–98.8 %) | Ref | (<0.001) |
|  | 2 cm to less than 5 cm | 1423 (185) | 94.7% (93.4–95.8 %) | 2.16 (1.58 – 2.97) | <0.001 |
|  | ≥5 cm | 286 (79) | 85.2% (80.4–88.9 %) | 5.12 (3.57 – 7.32) | <0.001 |
| Number of pathological lymph node metastases | 0 | 1317 (92) | 97.1% (96.0–97.9 %) | Ref | (<0.001) |
|  | 1-3 | 995 (143) | 94.2% (92.6–95.5 %) | 2.09 (1.61 – 2.72) | <0.001 |
|  | ≥4 | 404 (122) | 85.6% (81.7–88.7 %) | 4.86 (3.72 – 6.39) | <0.001 |
| NAC | No | 2103 (208) | 96.2% (95.3–97.0 %) | Ref | <0.001 |
|  | Yes | 628 (153) | 87.5% (84.6–89.9 %) | 2.71 (2.20 – 3.34) |  |
| Pathological therapeutic response | Grade 0 | 42 (14) | 73.4% (57.1–84.3 %) | Ref | (0.012) |
|  | Grade 1 | 454 (116) | 88.3% (84.9–91.0 %) | 0.66 (0.38 – 1.15) | 0.143 |
|  | Grade 2 | 154 (30) | 88.8% (82.6–92.9 %) | 0.49 (0.26 – 0.93) | 0.028 |
|  | Grade 3 | 50 (4) | 97.9% (86.1–99.7 %) | 0.188 (0.062 – 0.571) | 0.003 |

*1 Wald test p-value for HR

*2 Number of parentheses represents p-value for the comparison among all categories.

Abbreviations: DRFS, distant recurrence-free survival; CI, confidential interval; HR, hazard ratio; NAC, neoadjuvant chemotherapy

**Supplementary Table 3** Prognostic factors for OS in the univariable analysis

| Factor | Level | n (event) | HR (95% CI) | *p* value^*1^ |
| --- | --- | --- | --- | --- |
| Age | 20-39 years | 274 (23) | Ref | (<0.001)^*2^ |
|  | 40-69 yeras | 2094 (126) | 0.69 (0.45 – 1.08) | 0.107 |
|  | ≥70 years | 364 (45) | 1.98 (1.20 – 3.29) | 0.008 |
| Menopausal status | Premenopausal | 1395 (75) | Ref | <0.001 |
|  | Postmenopausal | 1311 (118) | 1.84 (1.38 – 2.46) |  |
| Bilateral breast cancer | No | 2690 (187) | Ref | 0.018 |
|  | Yes | 42 (7) | 2.49 (1.17 – 5.30) |  |
| ER | <1% | 15 (2) | Ref | (0.049) |
|  | 1-9% | 18 (3) | 1.61 (0.27 – 9.62) | 0.604 |
|  | ≥10% | 1722 (134) | 0.68 (0.17 – 2.75) | 0.589 |
|  | Unknown | 977 (55) | 0.48 (0.12 – 1.97) | 0.309 |
| PgR | <1% | 152 (17) | Ref | (0.038) |
|  | ≥1% | 825 (63) | 0.68 (0.40 – 1.17) | 0.162 |
|  | Unknown | 1755 (114) | 0.54 (0.33 – 0.90) | 0.018 |
| HER2 | 0 | 1386 (107) | Ref | (0.411) |
|  | 1+ | 1008 (69) | 0.89 (0.66 – 1.21) | 0.453 |
|  | 2+ | 338 (18) | 0.73 (0.44 – 1.20) | 0.212 |
| Ki-67 | <14% | 371 (21) | Ref | (0.001) |
|  | 14-29% | 315 (23) | 1.73 (0.73 – 2.39) | 0.352 |
|  | ≥30% | 367 (42) | 2.21 (1.31 – 3.74) | 0.003 |
|  | Unknown | 1679 (108) | 1.12 (0.70 – 1.79) | 0.631 |
| Nuclear grade | Grade 1 | 1041 (38) | Ref | (<0.001) |
|  | Grade 2 | 1039 (86) | 2.17 (1.48 – 3.18) | <0.001 |
|  | Grade 3 | 456 (43) | 2.50 (1.62 – 3.87) | <0.001 |
| Histological grade | Grade 1 | 149 (9) | Ref | (0.288) |
|  | Grade 2 | 218 (21) | 1.48 (0.68 – 3.25) | 0.322 |
|  | Grade 3 | 69 (12) | 2.03 (0.84 – 4.90) | 0.114 |
| Lymphatic invasion | No | 1545 (89) | Ref | 0.001 |
|  | Yes | 1166 (104) | 1.59 (1.20 – 2.11) |  |
| Vascular invasion | No | 2427 (165) | Ref | 0.071 |
|  | Yes | 284 (28) | 1.48 (0.97 – 2.16) |  |
| Pathological tumor size | <2 cm | 765 (22) | Ref | (<0.001) |
|  | 2 cm to less than 5 cm | 1423 (97) | 2.42 (1.52 – 3.84) | <0.001 |
|  | ≥5 cm | 286 (41) | 5.40 (3.22 – 9.06) | <0.001 |
| Number of pathological lymph node metastases | 0 | 1317 (47) | Ref | (<0.001) |
|  | 1-3 | 995 (82) | 2.28 (1.59 – 3.26) | <0.001 |
|  | ≥4 | 404 (63) | 4.54 (3.11 – 6.63) | <0.001 |
| NAC | No | 2103 (101) | Ref | <0.001 |
|  | Yes | 628 (92) | 3.22 (2.43 – 4.27) |  |
| Pathological therapeutic response | Grade 0 | 42 (12) | Ref | (0.008) |
|  | Grade 1 | 454 (68) | 0.44 (0.24 – 0.82) | 0.003 |
|  | Grade 2 | 154 (20) | 0.39 (0.19 – 0.80) | 0.010 |
|  | Grade 3 | 50 (2) | 0.11 (0.03 – 0.50) | 0.004 |

*1 Wald test p-value for HR

*2 Number of parentheses represents p-value for the comparison among all categories.

Abbreviations: OS, overall survival; CI, confidential interval; HR, hazard ratio; NAC, neoadjuvant chemotherapy

**Supplementary Table 4a** Prognostic factors for cumulative recurrence rate in univariable analysis

| Factor | Level | n (event） | 3-year cumulative recurrence rate (95%CI) | HR (95% CI) | *p* value^*1^ |
| --- | --- | --- | --- | --- | --- |
| Age | 20-39 years | 274 (63) | 14.0% (10.2–18.5 %) | Ref | (<0.001)^*2^ |
|  | 40-69 years | 2094 (288) | 5.9% (4.9–7.0 %) | 0.55 (0.42 – 0.72) | <0.001 |
|  | ≥70 years | 364 (44) | 6.1% (3.9–8.9 %) | 0.57 (0.39 – 0.84) | 0.005 |
| Menopausal status | Premenopausal | 1395 (212) | 7.6% (6.3–9.1 %) | Ref | 0.602 |
|  | Postmenopausal | 1311 (182) | 5.8% (4.6–7.2 %) | 0.950 (0.78 – 1.16) |  |
| Bilateral breast cancer | No | 2690 (386) | 6.6% (5.7–7.6 %) | Ref | 0.211 |
|  | Yes | 42 (9) | 14.3% (5.7–26.6 %) | 1.53 (0.79 – 2.97) |  |
| ER | <1% | 15 (2) | 6.7% (0.4–26.9 %) | Ref | (0.525) |
|  | 1-9% | 18 (4) | 23.0% (6.7–44.8 %) | 2.18 (0.39 – 12.93) | 0.379 |
|  | ≥10% | 1722 (230) | 6.2% (5.1–7.4 %) | 1.18 (0.30 – 4.69) | 0.810 |
|  | Unknown | 977 (150) | 7.4% (5.8–9.1 %) | 1.32 (0.33 – 5.23) | 0.696 |
| PgR | <1% | 152 (23) | 8.1% (4.4–13.2 %) | Ref | (0.680) |
|  | ≥1% | 825 (123) | 6.3% (4.7–8.1 %) | 0.99 (0.64 – 1.55) | 0.974 |
|  | Unknown | 1755 (249) | 6.8% (5.7–8.1 %) | 0.91 (0.59 – 1.39) | 0.655 |
| HER2 | 0 | 1386 (204) | 6.9% (5.7–8.4 %) | Ref | (0.880) |
|  | 1+ | 1008 (142) | 6.4% (4.9–8.0 %) | 0.96 (0.78 – 1.19) | 0.736 |
|  | 2+ | 338 (49) | 7.1% (4.6–10.2 %) | 1.04 (0.76 – 1.43) | 0.789 |
| Ki-67 | <14% | 371 (39) | 4.4% (2.6–6.9 %) | Ref | (<0.001) |
|  | 14-29% | 315 (52) | 7.0% (4.5–10.2 %) | 1.66 (1.20 – 2.50) | 0.017 |
|  | ≥30% | 367 (80) | 12.4% (9.2–16.0 %) | 2.32 (1.58 – 3.41) | <0.001 |
|  | Unknown | 1679 (224) | 6.0% (4.9–7.2 %) | 1.27 (0.91 – 1.79) | 0.164 |
| Nuclear grade | Grade 1 | 1041 (83) | 3.7% (2.6–5.0 %) | Ref | (<0.001) |
|  | Grade 2 | 1039 (188) | 7.6% (6.1–9.4 %) | 2.29 (1.77 – 2.97) | <0.001 |
|  | Grade 3 | 456 (83) | 10.3% (7.7–13.3 %) | 2.35 (1.73 – 3.19) | <0.001 |
| Histological grade | Grade 1 | 149 (19) | 6.1% (3.0–10.8 %) | Ref | (0.140) |
|  | Grade 2 | 218 (44) | 8.8% (5.5–13.1 %) | 1.64 (0.96 – 2.80) | 0.073 |
|  | Grade 3 | 69 (17) | 13.2% (6.4–22.4 %) | 1.80 (0.92 – 3.51) | 0.085 |
| Lymphatic invasion | No | 1545 (160) | 4.5% (3.5–5.6 %) | Ref | <0.001 |
|  | Yes | 1166 (234) | 9.8% (8.1–11.6 %) | 2.07 (1.70 – 2.53) |  |
| Vascular invasion | No | 2427 (337) | 6.6% (5.6–7.6 %) | Ref | 0.005 |
|  | Yes | 284 (57) | 8.1% (5.2–11.7 %) | 1.49 (1.13 – 1.97) |  |
| Pathological tumor size | <2 cm | 765 (58) | 2.8% (1.8–4.2 %) | Ref | (<0.001) |
|  | 2 cm to less than 5 cm | 1423 (210) | 6.4% (5.2–7.8 %) | 2.03 (1.52 – 2.71) | <0.001 |
|  | ≥5 cm | 286 (80) | 15.9% (11.8–20.4 %) | 4.24 (3.02 – 5.95) | <0.001 |
| Number of pathological lymph node metastases | 0 | 1317 (117) | 3.8% (2.9–5.0 %) | Ref | (<0.001) |
|  | 1-3 | 995 (150) | 6.7% (5.2–8.4 %) | 1.72 (1.35 – 2.18) | <0.001 |
|  | ≥4 | 404 (124) | 15.4% (12.1–19.2 %) | 3.86 (3.00 – 4.97) | <0.001 |
| NAC | No | 2103 (239) | 4.8% (3.9–5.8 %) | Ref | <0.001 |
|  | Yes | 628 (155) | 13.1% (10.6–15.9 %) | 2.38 (1.94 – 2.92) |  |
| Pathological therapeutic response | Grade 0 | 42 (15) | 31.5% (18.0–45.9 %) | Ref | (0.011) |
|  | Grade 1 | 454 (114) | 12.1% (9.3–15.4 %) | 0.59 (0.33 – 1.04) | 0.070 |
|  | Grade 2 | 154 (35) | 13.2% (8.4–19.1 %) | 0.53 (0.28 – 1.00) | 0.051 |
|  | Grade 3 | 50 (3) | 2.1% (0.2–9.7 %) | 0.13 (0.04 – 0.44) | 0.001 |

*1 Wald test p-value for HR with Fine & Gray model

*2 Number of parentheses represents p-value for the comparison among all categories.

Abbreviations: CI, confidence interval; HR, hazard ratio; NAC, neoadjuvant chemotherapy

**Supplementary Table 4b** Prognostic factors for cumulative recurrence rate in multivariable analysis

| Factor | Level | n (event)^*1^ | Including all covariates | |
| --- | --- | --- | --- | --- |
|  |  |  | HR (95% CI) | *p* value^*2^ |
| Age | 20-39 years | 233 (51) | Ref | (0.027)^*3^ |
|  | 40-69 years | 1739 (237) | 0.63 (0.45 – 0.88) | 0.008 |
|  | ≥70 years | 337 (37) | 0.67 (0.40 – 1.11) | 0.116 |
| Menopausal status | Premenopausal | 1172 (175) | Ref | 0.396 |
|  | Postmenopausal | 1137 (150) | 1.12 (0.86 – 1.47) |  |
| Bilateral breast cancer | No | 2272 (316) | Ref | 0.131 |
|  | Yes | 37 (9) | 1.74 (0.85 – 3.57) |  |
| ER | <1% | 15 (2) | Ref | (0.960) |
|  | 1-9% | 10 (2) | 1.36 (0.15 – 12.08) | 0.781 |
|  | ≥10% | 1378 (182) | 1.35 (0.33 – 5.53) | 0.674 |
|  | Unknown | 906 (139) | 1.44 (0.34 – 6.12) | 0.621 |
| PgR | <1% | 137 (23) | Ref | (0.901) |
|  | ≥1% | 751 (110) | 0.96 (0.57 – 1.62) | 0.867 |
|  | Unknown | 1421 (192) | 0.89 (0.50 – 1.59) | 0.688 |
| HER2 | 0 | 1179 (172) | Ref | (0.701) |
|  | 1+ | 817 (109) | 0.99 (0.77 – 1.28) | 0.956 |
|  | 2+ | 313 (44) | 0.86 (0.61 – 1.22) | 0.411 |
| Ki-67 | <14% | 358 (38) | Ref | (0.841) |
|  | 14-29% | 299 (52) | 1.06 (0.67 – 1.68) | 0.804 |
|  | ≥30% | 321 (73) | 1.16 (0.73 – 1.87) | 0.519 |
|  | Unknown | 1331 (162) | 1.01 (0.68 – 1.50) | 0.980 |
| Nuclear grade | Grade 1 | 958 (73) | Ref | (<0.001) |
|  | Grade 2 | 953 (174) | 1.95 (1.45 – 2.61) | <0.001 |
|  | Grade 3 | 398 (78) | 1.83 (1.28 – 2.62) | 0.001 |
| Lymphatic invasion | No | 1263 (117) | Ref | 0.004 |
|  | Yes | 1046 (208) | 1.52 (1.14 – 2.02) |  |
| Vascular invasion | No | 2035 (269) | Ref | 0.080 |
|  | Yes | 274 (56) | 1.33 (0.97 – 1.83) |  |
| Pathological tumor size | <2 cm | 704 (51) | Ref | (<0.001) |
|  | 2 cm to less than 5 cm | 1334 (200) | 2.06 (1.50 – 2.83) | <0.001 |
|  | ≥5 cm | 271 (74) | 2.39 (1.61 – 3.56) | <0.001 |
| Number of pathological lymph node metastases | 0 | 1154 (106) | Ref | (0.002) |
|  | 1-3 | 837 (126) | 1.12 (0.83 – 1.51) | 0.474 |
|  | ≥4 | 318 (93) | 1.76 (1.23 – 2.51) | 0.002 |
| NAC | No | 1994 (226) | Ref | <0.001 |
|  | Yes | 315 (99) | 2.35 (1.76 – 3.14) |  |

*1 Analysis was performed on the 2309 cases for which all explanatory variables were obtained.

*2 Wald test p-value for HR

*3 Number of parentheses represents p-value for the comparison among all categories.

Abbreviations: CI, confidence interval; HR, hazard ratio; NAC, neoadjuvant chemotherapy

**Supplementary Table 5a** Prognostic factors for cumulative distant metastasis rate in univariable analysis

| Factor | Level | n (event) | 3-year cumulative distant metastasis rate (95% CI) | HR (95% CI) | *p* value^*1^ |
| --- | --- | --- | --- | --- | --- |
| Age | 20-39 years | 274 (51) | 10.7% (7.3–14.8 %) | Ref | (0.001)^*2^ |
|  | 40-69 years | 2094 (232) | 4.8% (3.9–5.8 %) | 0.56 (0.41 – 0.76) | <0.001 |
|  | ≥70 years | 364 (34) | 4.3% (2.5–6.8 %) | 0.56 (0.36 – 0.86) | 0.008 |
| Menopausal status | Premenopausal | 1395 (171) | 6.0% (4.8–7.4 %) | Ref | 0.608 |
|  | Postmenopausal | 1311 (145) | 4.6% (3.5–5.8 %) | 0.94 (0.76 – 1.18) |  |
| Bilateral breast cancer | No | 2690 (308) | 5.2% (4.4–6.1 %) | Ref | 0.047 |
|  | Yes | 42 (9) | 14.3% (5.7–26.6 %) | 1.97 (1.01 – 3.83) |  |
| ER | <1% | 15 (2) | 6.7% (0.4–26.9 %) | Ref | (0.500) |
|  | 1-9% | 18 (4) | 23.0% (6.7–44.8 %) | 2.21 (0.39 – 12.48) | 0.371 |
|  | ≥10% | 1722 (201) | 5.1% (4.1–6.3 %) | 0.99 (0.25 – 3.93) | 0.993 |
|  | Unknown | 977 (110) | 5.3% (4.0–6.8 %) | 0.95 (0.24 – 3.78) | 0.942 |
| PgR | <1% | 152 (21) | 7.5% (3.9–12.4 %) | Ref | (0.116) |
|  | ≥1% | 825 (106) | 5.5% (4.1–7.2 %) | 0.94 (0.58 – 1.50) | 0.792 |
|  | Unknown | 1755 (190) | 5.0% (4.1–6.1 %) | 0.75 (0.48 – 1.18) | 0.210 |
| HER2 | 0 | 1386 (167) | 5.7% (4.5–7.0 %) | Ref | (0.357) |
|  | 1+ | 1008 (107) | 4.5% (3.3–5.9 %) | 0.88 (0.69 – 1.13) | 0.313 |
|  | 2+ | 338 (43) | 6.2% (3.9–9.1 %) | 1.13 (0.81 – 1.58) | 0.486 |
| Ki-67 | <14% | 371 (28) | 3.3% (1.8–5.6 %) | Ref | (<0.001) |
|  | 14-29% | 315 (47) | 5.7% (3.4–8.7 %) | 2.08 (1.31 – 3.32) | 0.002 |
|  | ≥30% | 367 (68) | 11.3% (8.2–14.8 %) | 2.73 (1.75 – 4.25) | <0.001 |
|  | Unknown | 1679 (174) | 4.4% (3.5–5.5 %) | 1.37 (0.92 – 2.04) | 0.123 |
| Nuclear grade | Grade 1 | 1041 (62) | 2.3% (1.5–3.4 %) | Ref | (<0.001) |
|  | Grade 2 | 1039 (151) | 6.4% (5.0–8.1 %) | 2.44 (1.82 – 3.28) | <0.001 |
|  | Grade 3 | 456 (70) | 8.5% (6.2–11.3 %) | 2.59 (1.84 – 3.66) | <0.001 |
| Histological grade | Grade 1 | 149 (18) | 5.4% (2.5–9.9 %) | Ref | (0.203) |
|  | Grade 2 | 218 (36) | 7.0% (4.1–10.9 %) | 1.38 (0.78 – 2.42) | 0.269 |
|  | Grade 3 | 69 (17) | 13.2% (6.4–22.4 %) | 1.85 (0.94 – 3.64) | 0.075 |
| Lymphatic invasion | No | 1545 (130) | 3.4% (2.6–4.4 %) | Ref | <0.001 |
|  | Yes | 1166 (186) | 7.8% (6.4–9.5 %) | 2.00 (1.60 – 2.50) |  |
| Vascular invasion | No | 2427 (268) | 5.2% (4.4–6.1 %) | Ref | 0.004 |
|  | Yes | 284 (48) | 6.3% (3.8–9.6 %) | 1.56 (1.15 – 2.12) |  |
| Pathological tumor size | <2 cm | 765 (38) | 1.8% (1.0–2.9 %) | Ref | (<0.001) |
|  | 2 cm to less than 5 cm | 1423 (162) | 4.7% (3.7–5.9 %) | 2.38 (1.68 – 3.38) | <0.001 |
|  | ≥5 cm | 286 (74) | 14.4% (10.6–18.9 %) | 5.98 (4.05 – 8.84) | <0.001 |
| Number of pathological lymph node metastases | 0 | 1317 (74) | 2.2% (1.5–3.1 %) | Ref | (<0.001) |
|  | 1-3 | 995 (125) | 5.7% (4.3–7.3 %) | 2.28 (1.71 – 3.08) | <0.001 |
|  | ≥4 | 404 (115) | 13.9% (10.7–17.6 %) | 5.68 (4.25 – 7.60) | <0.001 |
| NAC | No | 2103 (174) | 3.3% (2.6–4.1 %) | Ref | <0.001 |
|  | Yes | 628 (142) | 12.0% (9.6–14.8 %) | 2.99 (2.40 – 3.74) |  |
| Pathological therapeutic response | Grade 0 | 42 (12) | 26.6% (14.2–40.8 %) | Ref | (0.016) |
|  | Grade 1 | 454 (108) | 11.2% (8.5–14.4 %) | 0.73 (0.39 – 1.38) | 0.334 |
|  | Grade 2 | 154 (26) | 10.6% (6.3–16.1 %) | 0.50 (0.25 – 1.03) | 0.061 |
|  | Grade 3 | 50 (3) | 2.1% (0.2–9.7 %) | 0.17 (0.05 – 0.61) | 0.006 |

*1 Wald test p-value for HR with Fine & Gray model

*2 Number of parentheses represents p-value for the comparison among all categories.

Abbreviations: CI, confidence interval; HR, hazard ratio; NAC, neoadjuvant chemotherapy

**Supplementary Table 5b** Prognostic factors for cumulative distant metastasis rate in multivariable analysis

| Factor | Level | n (event)^*1^ | Including all covariates | |
| --- | --- | --- | --- | --- |
|  |  |  | HR (95% CI) | *p* value^*2^ |
| Age | 20-39 years | 233 (40) | Ref | (0.078)^*3^ |
|  | 40-69 years | 1739 (187) | 0.65 (0.44 – 0.95) | 0.026 |
|  | ≥70 years | 337 (29) | 0.71 (0.40 – 1.25) | 0.235 |
| Menopausal status | Premenopausal | 1172 (138) | Ref | 0.604 |
|  | Postmenopausal | 1137 (118) | 1.08 (0.80 – 1.47) |  |
| Bilateral breast cancer | No | 2272 (247) | Ref | 0.068 |
|  | Yes | 37 (9) | 2.07 (0.95 – 4.53) |  |
| ER | <1% | 15 (2) | Ref | (0.896) |
|  | 1-9% | 10 (2) | 1.21 (0.12 – 12.02) | 0.870 |
|  | ≥10% | 1378 (152) | 1.08 (0.26 – 4.53) | 0.918 |
|  | Unknown | 906 (100) | 0.92 (0.21 – 4.05) | 0.914 |
| PgR | >1% | 137 (21) | Ref | (0.851) |
|  | ≥1% | 751 (94) | 0.93 (0.52 – 1.66) | 0.677 |
|  | Unknown | 1421 (141) | 0.84 (0.45 – 1.59) | 0.842 |
| HER2 | 0 | 1179 (139) | Ref | (0.868) |
|  | 1+ | 817 (79) | 0.94 (0.70 – 1.26) | 0.677 |
|  | 2+ | 313 (38) | 1.04 (0.71 – 1.52) | 0.842 |
| Ki-67 | <14% | 358 (27) | Ref | (0.540) |
|  | 14-29% | 299 (47) | 1.40 (0.83 – 2.37) | 0.205 |
|  | ≥30% | 321 (61) | 1.47 (0.84 – 2.57) | 0.173 |
|  | Unknown | 1331 (121) | 1.27 (0.79 – 2.05) | 0.329 |
| Nuclear grade | Grade 1 | 958 (53) | Ref | (<0.001) |
|  | Grade 2 | 953 (139) | 1.99 (1.41 – 2.80) | <0.001 |
|  | Grade 3 | 398 (64) | 1.90 (1.26 – 2.87) | 0.002 |
| Lymphatic invasion | No | 1263 (91) | Ref | 0.127 |
|  | Yes | 1046 (165) | 1.28 (0.93 – 1.75) |  |
| Vascular invasion | No | 2035 (209) | Ref | 0.165 |
|  | Yes | 274 (47) | 1.29 (0.90 – 1.85) |  |
| Pathological tumor size | <2 cm | 704 (33) | Ref | (<0.001) |
|  | 2 cm to less than 5 cm | 1334 (154) | 2.47 (1.68 – 3.63) | <0.001 |
|  | ≥5 cm | 271 (69) | 3.11 (1.95 – 4.95) | <0.001 |
| Number of pathological lymph node metastases | 0 | 1154 (66) | Ref | (<0.001) |
|  | 1-3 | 837 (103) | 1.54 (1.09 – 2.18) | 0.021 |
|  | ≥4 | 318 (87) | 2.67 (1.79 – 4.00) | <0.001 |
| NAC | No | 1994 (165) | Ref | <0.001 |
|  | Yes | 315 (91) | 2.63 (1.91 – 3.62) |  |

*1 Analysis was performed on the 2309 cases for which all explanatory variables were obtained.

*2 Wald test p-value for HR

*3 Number of parentheses represents p-value for the comparison among all categories.

Abbreviations: CI, confidence interval; HR, hazard ratio; NAC, neoadjuvant chemotherapy

**Supplementary Table 6** Characteristics of patient population meeting eligibility criteria for monarchE trial

| Characteristics | WJOG15721B  eligible patients  (n=2732) | monarchE ITT population  (n=769) | monarchE  Cohort 1  (n=673) | monarchE  Cohort 2  (n=96) |
| --- | --- | --- | --- | --- |
| Age, years |  |  |  |  |
| Median (range) | 51 (23-96) | 50 (25-93) | 50 (25-93) | 50 (25-93) |
| Performance status |  |  |  |  |
| 0 | 1318 (48.2) | 376 (48.9) | 318 (47.3) | 58 (60.4) |
| 1 | 16 (0.6) | 3 (0.4) | 3 (0.4) | 0 (0) |
| Unknown | 1398 (51.2) | 390 (50.7) | 352 (52.3) | 38 (39.6) |
| Comorbidity |  |  |  |  |
| None | 1057 (38.7) | 344 (44.7) | 295 (43.8) | 49 (51.0) |
| Hypertension | 219 (8.0) | 68 (8.8) | 57 (8.5) | 11 (11.5) |
| Diabetes | 87 (3.2) | 27 (3.5) | 23 (3.4) | 4 (4.2) |
| Malignant disease except breast cancer | 59 (2.2) | 15 (2.0) | 14 (2.1) | 1 (1.0) |
| Cardiac disease | 16 (0.6) | 4 (0.5) | 4 (0.6) | 0 (0) |
| Cerebrovascular and peripheral vascular disease | 8 (0.3) | 4 (0.5) | 4 (0.6) | 0 (0) |
| Collagen disease | 8 (0.3) | 2 (0.3) | 1 (0.1) | 1 (1.0) |
| Chronic liver disease | 5 (0.2) | 2 (0.3) | 2 (0.3) | 0 (0) |
| Renal dysfunction | 4 (0.1) | 2 (0.3) | 2 (0.3) | 0 (0) |
| Other | 405 (14.8) | 96 (12.5) | 79 (11.7) | 17 (17.7) |
| Unknown | 1004 (36.7) | 205 (26.7) | 192 (28.5) | 13 (13.5) |
| Menopausal status |  |  |  |  |
| Premenopausal | 1395 (51.1) | 410 (53.3) | 356 (52.9) | 54 (56.3) |
| Postmenopausal | 1311 (48.0) | 361 (46.9) | 309 (45.9) | 52 (54.2) |
| Unknown | 26 (1.0) | 8 (1.0) | 8 (1.2) | 0 (0) |
| Diagnostic occasion |  |  |  |  |
| Symptom awareness | 2005 (73.4) | 597 (77.6) | 524 (77.9) | 73 (76.0) |
| Detection by medical checkup | 404 (14.8) | 98 (12.7) | 89 (13.2) | 9 (9.4) |
| Accidental detection | 94 (3.4) | 23 (3.0) | 20 (3.0) | 3 (3.1) |
| Other | 229 (8.4) | 51 (6.6) | 40 (5.9) | 11 (11.5) |
| Bilateral breast cancer |  |  |  |  |
| No | 2690 (98.5) | 751 (97.7) | 658 (97.8) | 93 (96.9) |
| Yes | 42 (1.5) | 18 (2.3) | 15 (2.2) | 3 (3.1) |

**Supplementary Table 7** Clinicopathological features of patient population meeting eligibility criteria for monarchE trial

|  | WJOG15721B  eligible patients  (n=2732) | monarchE ITT population  (n=769) | monarchE  Cohort 1  (n=673) | monarchE  Cohort 2  (n=96) |
| --- | --- | --- | --- | --- |
| Clinical T factor |  |  |  |  |
| Tis | 2 (0.1) | 0 (0) | 0 (0) | 0 (0) |
| T1 | 193 (7.1) | 87 (11.3) | 75 (11.1) | 12 (12.5) |
| T2 | 2205 (80.7) | 507 (65.9) | 431 (64.0) | 76 (79.2) |
| T3 | 197 (7.2) | 109 (14.2) | 103 (15.3) | 6 (6.3) |
| T4 | 135 (4.9) | 66 (8.6) | 64 (9.5) | 2 (2.1) |
| Clinical N factor |  |  |  |  |
| N0 | 1781 (65.2) | 254 (33.0) | 212 (31.5) | 42 (43.8) |
| N1 | 775 (28.4) | 391 (50.9) | 346 (51.4) | 45 (46.9) |
| N2 | 88 (3.2) | 58 (7.5) | 53 (7.9) | 5 (5.2) |
| N3 | 88 (3.2) | 66 (8.6) | 62 (9.2) | 4 (4.2) |
| Clinical stage |  |  |  |  |
| IIA | 1841 (67.4) | 293 (38.1) | 242 (36.0) | 51 (53.1) |
| IIB | 529 (19.4) | 254 (33.0) | 223 (33.1) | 31 (32.3) |
| IIIA | 160 (5.9) | 105 (13.7) | 97 (14.4) | 8 (8.3) |
| IIIB | 114 (4.2) | 51 (6.6) | 49 (7.3) | 2 (2.1) |
| IIIC | 88 (3.2) | 66 (8.6) | 62 (9.2) | 4 (4.2) |
| Pathological T factor |  |  |  |  |
| T0 | 18 (0.7) | 2 (0.3) | 2 (0.3) | 0 (0) |
| Tis | 23 (0.8) | 2 (0.3) | 1 (0.1) | 1 (1.0) |
| T1 | 864 (31.6) | 145 (18.9) | 114 (16.9) | 31 (32.3) |
| T2 | 1290 (47.2) | 321 (41.7) | 257 (38.2) | 64 (66.7) |
| T3 | 257 (9.4) | 201 (26.1) | 201 (29.9) | 0 (0) |
| T4 | 32 (1.2) | 17 (2.2) | 17 (2.5) | 0 (0) |
| Unknown | 248 (9.1) | 81 (10.5) | 81 (12.0) | 0 (0) |
| Pathological N factor |  |  |  |  |
| N0 | 1323 (48.4) | 0 (0) | 0 (0) | 0 (0) |
| N1 | 995 (36.4) | 366 (47.6) | 270 (40.1) | 96 (100) |
| N2 | 281 (10.3) | 280 (36.4) | 280 (41.6) | 0 (0) |
| N3 | 124 (4.5) | 123 (16.0) | 123 (18.3) | 0 (0) |
| Unknown | 9 (0.3) | 0 (0) | 0 (0) | 0 (0) |
| Pathological stage |  |  |  |  |
| 0 | 19 (0.7) | 0 (0) | 0 (0) | 0 (0) |
| I | 519 (19.0) | 5 (0.7) | 0 (0) | 5 (5.2) |
| IIA | 975 (35.7) | 75 (9.8) | 48 (7.1) | 27 (28.1) |
| IIB | 593 (21.8) | 177 (23.0) | 113 (16.8) | 64 (66.7) |
| IIIA | 374 (13.7) | 373 (48.5) | 373 (55.4) | 0 (0) |
| IIIB | 29 (1.1) | 15 (2.0) | 15 (2.2) | 0 (0) |
| IIIC | 124 (4.5) | 123 (16.0) | 123 (18.3) | 0 (0) |
| Unknown | 99 (3.6) | 1 (0.1) | 1 (0.1) | 0 (0) |
| Histological type |  |  |  |  |
| Invasive cancer | 12 (0.4) | 6 (0.8) | 5 (0.7) | 1 (1.0) |
| Invasive ductal carcinoma | 2305 (84.3) | 670 (87.1) | 581 (86.3) | 89 (92.7) |
| Special type | 402 (14.7) | 90 (11.7) | 85 (12.6) | 5 (5.2) |
| Invasive lobular carcinoma | 165 (6.0) | 55 (7.2) | 53 (7.9) | 2 (2.1) |
| Mucinous carcinoma | 153 (5.6) | 12 (1.6) | 11 (1.6) | 1 (1.0) |
| Invasive micropapillary carcinoma | 38 (1.4) | 16 (2.1) | 16 (2.4) | 0 (0) |
| Other | 46 (1.7) | 7 (0.9) | 5 (0.7) | 2 (2.1) |
| Mixed type | 6 (0.2) | 2 (0.3) | 1 (0.1) | 1 (1.0) |
| Unknown | 7 (0.3) | 1 (0.1) | 1 (0.1) | 0 (0) |
| Nuclear grade |  |  |  |  |
| 1 | 1041 (38.1) | 176 (22.9) | 156 (23.2) | 20 (20.8) |
| 2 | 1039 (38.0) | 297 (38.7) | 221 (32.8) | 76 (79.2) |
| 3 | 456 (16.7) | 245 (31.9) | 245 (36.4) | 0 (0) |
| Unknown | 196 (7.2) | 51 (6.6) | 51 (7.6) | 0 (0) |
| Histological grade |  |  |  |  |
| 1 | 149 (5.5) | 28 (3.6) | 20 (3.0) | 8 (8.3) |
| 2 | 218 (8.0) | 71 (9.2) | 54 (8.0) | 17 (17.7) |
| 3 | 69 (2.6) | 37 (4.8) | 33 (4.9) | 4 (4.2) |
| Unknown | 2301 (84.2) | 633 (82.3) | 566 (84.1) | 67 (69.8) |
| ER |  |  |  |  |
| <1% | 15 (0.5) | 5 (0.7) | 5 (0.7) | 0 (0) |
| 1-9% | 18 (0.7) | 8 (1.0) | 8 (1.2) | 0 (0) |
| ≥10% | 1722 (63.0) | 495 (64.4) | 429 (63.7) | 66 (68.8) |
| Unknown | 977 (35.8) | 251 (32.6) | 231 (34.3) | 30 (31.3) |
| PgR |  |  |  |  |
| <1% | 152 (5.6) | 53 (6.9) | 48 (7.1) | 5 (5.2) |
| ≥1% | 825 (30.2) | 272 (35.4) | 217 (32.2) | 55 (57.3) |
| Unknown | 1755 (64.2) | 444 (57.7) | 408 (60.6) | 36 (37.5) |
| HER2 |  |  |  |  |
| 0 | 1386 (50.7) | 390 (50.7) | 347 (51.6) | 43 (44.8) |
| 1+ | 1008 (36.9) | 282 (36.7) | 251 (37.3) | 31 (32.3) |
| 2+ | 338 (12.4) | 97 (12.6) | 75 (11.1) | 22 (22.9) |
| Ki-67 |  |  |  |  |
| <14% | 371 (13.6) | 113 (14.7) | 71 (10.5) | 42 (43.8) |
| 14-29% | 315 (11.5) | 117 (15.2) | 69 (10.3) | 48 (50) |
| ≥30% | 367 (13.4) | 136 (17.7) | 130 (19.3) | 6 (6.25) |
| Unknown | 1679 (61.5) | 403 (52.4) | 403 (59.9) | 0 (0) |
| Lymphatic invasion |  |  |  |  |
| No | 1545 (56.6) | 239 (31.1) | 189 (28.1) | 40 (41.7) |
| Yes | 1166 (42.7) | 539 (70.1) | 483 (71.8) | 56 (58.3) |
| Unknown | 21 (0.8) | 1 (0.1) | 1 (0.1) | 0 (0) |
| Vascular invasion |  |  |  |  |
| No | 2427 (88.8) | 646 (84.0) | 564 (83.8) | 82 (85.4) |
| Yes | 284 (10.4) | 122 (15.9) | 108 (16.0) | 14 (14.6) |
| Unknown | 21 (0.8) | 1 (0.1) | 1 (0.1) | 0 (0) |
| Pathological tumor size (cm) |  |  |  |  |
| <2 | 765 (28.0) | 119 (15.5) | 93 (13.8) | 26 (27.1) |
| 2 to less than 5 | 1423 (52.1) | 347 (45.1) | 277 (41.2) | 70 (72.9) |
| ≥5 | 286 (10.5) | 222 (28.9) | 222 (33.0) | 0 (0) |
| Unknown | 258 (9.4) | 0 (0) | 0 (0) | 0 (0) |
| Number of pathological lymph node metastases |  |  |  |  |
| 0 | 1317 (48.2) | 0 (0) | 0 (0) | 0 (0) |
| 1-3 | 995 (36.4) | 365 (47.5) | 269 (40.0) | 96 (0) |
| 4-9 | 280 (10.2) | 280 (36.4) | 280 (41.6) | 0 (0) |
| ≥10 | 124 (4.5) | 124 (16.1) | 124 (18.4) | 0 (0) |
| Unknown | 16 (0.6) | 0 (0) | 0 (0) | 0 (0) |
| Pathological therapeutic response^*1^ |  |  |  |  |
| Grade 0 | 42 (5.7) | 21 (6.8) | 19 (6.9) | 2 (5.9) |
| Grade 1 | 454 (61.6) | 198 (64.3) | 179 (65.3) | 19 (55.9) |
| Grade 2 | 154 (20.9) | 72 (23.4) | 60 (21.9) | 12 (35.3) |
| Grade 3 | 50 (6.8) | 7 (2.3) | 6 (2.2) | 1 (2.9) |
| Unknown | 37 (5.0) | 10 (3.2) | 10 (3.6) | 0 (0) |

*1 For Cohort 1, 274 patients who received preoperative chemotherapy or preoperative endocrine therapy were included. For Cohort 2, 34 patients who received preoperative chemotherapy or preoperative endocrine therapy were included.

Abbreviations: ER, estrogen receptor; PgR, progesterone receptor; HER2, human epidermal growth factor receptor 2

**Supplementary Table 8** Treatment details of patient population meeting eligibility criteria for monarchE trial

1. Surgical procedures and radiotherapy

|  | WJOG15721B  eligible patients  (n=2774^*1^) | monarchE ITT population  (n=787^*2^) | monarchE  Cohort 1  (n=688^*3^) | monarchE  Cohort 2  (n=99^*4^) |
| --- | --- | --- | --- | --- |
| Surgical procedure |  |  |  |  |
| Breast |  |  |  |  |
| Lumpectomy | 1043 (37.6) | 209 (26.6) | 165 (24.0) | 44 (44.4) |
| Mastectomy | 1731 (62.4) | 578 (73.4) | 523 (76.0) | 55 (55.6) |
| Sentinel lymph node biopsy |  |  |  |  |
| No | 935 (33.7) | 503 (63.9) | 451 (65.6) | 52 (52.5) |
| Yes | 1839 (66.3) | 84 (10.7) | 237 (34.4) | 47 (47.5) |
| Axillary lymph node dissection |  |  |  |  |
| No | 1452 (52.3) | 85 (10.8) | 60 (8.7) | 25 (25.3) |
| Yes | 1322 (47.7) | 702 (89.2) | 628 (91.3) | 74 (74.7) |
| Radiation therapy |  |  |  |  |
| No | 1222 (44.1) | 161 (20.5) | 129 (18.8) | 32 (32.3) |
| Yes | 1527 (55.0) | 621 (78.9) | 555 (80.7) | 66 (66.7) |
| Unknown | 25 (0.9) | 5 (0.6) | 4 (0.6) | 1 (1.0) |

*1 Total for all 2732 eligible cases, 2774 lesions including bilateral breast cancer.

*2, 3, 4 Similarly, tabulations are for 787 lesions, 688 lesions, and 99 lesions.

1. Neoadjuvant and adjuvant endocrine therapy or chemotherapy

|  | RealisE  eligible patients  (n=2732) | monarchE ITT population  (n=769) | monarchE  Cohort 1  (n=673) | monarchE  Cohort 2  (n=96) |
| --- | --- | --- | --- | --- |
| Neoadjuvant chemotherapy |  |  |  |  |
| No | 2104 (77.0) | 483 (62.8) | 421 (62.6) | 62 (64.6) |
| Yes | 628 (23.0) | 285 (37.1) | 251 (37.3) | 34 (35.4) |
| Anthracyclines | 617 (22.6) | 276 (35.9) | 246 (36.6) | 30 (31.3) |
| Taxanes | 628 (23.0) | 285 (37.1) | 251 (37.3) | 34 (35.4) |
| Others | 13 (0.5) | 7 (0.9) | 7 (1.0) | 0 (0) |
| Unknown | 1 (0.04) | 0 (0) | 0 (0) | 0 (0) |
| Neoadjuvant endocrine therapy |  |  |  |  |
| No | 2623 (96.0) | 746 (97.0) | 650 (96.6) | 96 (100) |
| Yes | 109 (4.0) | 23 (3.0) | 23 (3.4) | 0 (0) |
| SERM alone | 8 (0.3) | 2 (0.3) | 2 (0.3) | 0 (0) |
| SERM + LHRH agonist | 7 (0.3) | 1 (0.1) | 1 (0.1) | 0 (0) |
| AI alone | 88 (3.2) | 17 (2.2) | 17 (2.5) | 0 (0) |
| SERM + AI | 6 (0.2) | 3 (0.4) | 3 (0.4) | 0 (0) |
|  |  |  |  |  |
| Adjuvant chemotherapy |  |  |  |  |
| No | 1845 (67.5) | 366 (47.6) | 311 (46.2) | 55 (57.3) |
| Yes | 887 (32.5) | 403 (52.4) | 362 (53.8) | 41 (42.7) |
| CMF | 1 (0.04) | 1 (0.1) | 1 (0.1) | 0 (0) |
| Anthracyclines | 803 (29.4) | 365 (47.5) | 326 (48.4) | 39 (40.6) |
| Taxanes | 887 (32.5) | 403 (52.4) | 362 (53.8) | 41 (42.7) |
| Others | 68 (2.5) | 34 (4.4) | 31 (4.6) | 3 (3.1) |
| Adjuvant endocrine therapy |  |  |  |  |
| No | 0 (0) | 0 (0) | 0 (0) | 0 (0) |
| Yes | 2732 (100) | 769 (100) | 673 (100) | 96 (100) |
| SERM alone | 1002 (36.7) | 279 (36.3) | 246 (36.6) | 33 (34.4) |
| LHRH alone | 1 (0.04) | 0 (0) | 0 (0) | 0 (0) |
| AI alone | 1139 (41.7) | 325 (42.3) | 291 (43.2) | 34 (35.4) |
| SERM + LHRH agonist | 295 (10.8) | 78 (10.1) | 63 (9.4) | 15 (15.6) |
| SERM → AI | 177 (6.5) | 57 (7.4) | 51 (7.6) | 6 (6.3) |
| SERM + LHRH agonist → AI | 14 (0.5) | 4 (0.5) | 1 (0.1) | 3 (3.1) |
| AI + LHRH agonist | 6 (0.2) | 1 (0.1) | 0 (0) | 1 (1.0) |
| Others | 98 (3.6) | 0 (0) | 0 (0) | 0 (0) |
| Adjuvant endocrine therapy ≥5 years |  |  |  |  |
| No | 1612 (59.0) | 421 (54.7) | 373 (55.4) | 48 (50) |
| Yes | 1120 (41.0) | 348 (45.3) | 300 (44.6) | 48 (50) |

Abbreviations: SERM, selective estrogen receptor modulator; LHRH, luteinizing hormone-releasing hormone; AI, aromatase inhibitor; CMF, cyclophosphamide, methotrexate, and fluorouracil
